# Audit of therapeutic interventions in inpatient children using two scores: are they evidence-based in developing countries?

**DOI:** 10.1186/1472-6963-4-40

**Published:** 2004-12-29

**Authors:** Nilton Y Carreazo, Carlos A Bada, Juan P Chalco, Luis Huicho

**Affiliations:** 1 Universidad Nacional Mayor de San Marcos and Instituto de Salud del Niño, Lima, LI 05, Peru

## Abstract

**Background:**

The evidence base of clinical interventions in paediatric hospitals of developing countries has not been formally assessed. We performed this study to determine the proportion of evidence-based therapeutic interventions in a paediatric referral hospital of a developing country

**Methods:**

The medical records of 167 patients admitted in one-month period were revised. Primary diagnosis and primary therapeutic interventions were determined for each patient. A systematic search was performed to assess the level of evidence for each intervention. Therapeutic interventions were classified using the Ellis score and the Oxford Centre for Evidence Based Medicine Levels of Evidence

**Results:**

Any dehydration due to diarrhoea (59 cases) and pneumonia (42 cases) were the most frequent diagnoses. Based on Ellis score, level I evidence supported the primary therapeutic intervention in 21%, level II in 73% and level III in 6% cases. Using the Oxford classification 16%, 8%, 1% and 75% therapeutic interventions corresponded to grades A, B, C, and D recommendations, respectively. Overall, according to Ellis score, 94% interventions were evidence based. However, out of the total, 75% interventions were based on expert opinion or basic sciences. Most children with mild to moderate dehydration (52 cases) were inappropriately treated with slow intravenous fluids, and most children with non-complicated community acquired pneumonia (42 cases) received intravenous antibiotics

**Conclusions:**

Most interventions were inappropriate, despite the availability of effective therapy for several of them. Diarrhoeal dehydration and community acquired pneumonia were the most common diagnoses and were inappropriately managed. Existing effective interventions for dehydration and pneumonia need to be put into practice at referral hospitals of developing countries. For the remaining problems, there is the need to conduct appropriate clinical studies. Caution must be taken when assigning the level of evidence supporting therapeutic interventions, as commonly used classifications may be misleading

## Background

Previous studies have shown that medical interventions based on scientific evidence range from 10 to 80% [1-4]. These studies were performed in a broad spectrum of patients and settings in developed countries.

In paediatric practice, the proportion of evidence based interventions reported ranges from 75 to 91% [5-8]. These studies are clearly relevant to the quality of care in developed countries, with strong health systems, widely available high technology and qualified human resources. However, health services in developing countries are frequently weak, and they face too often severe lack of expensive technology. In addition, the level of qualification of health personnel may vary greatly within health facilities, even at referral level and in urban areas. Moreover, the prevalent childhood illnesses in developed countries are not necessarily those prevalent in developing countries.

The assessment of quality of care at referral level for severely ill children is an important component of the efforts for reducing the child mortality rate in poor countries, for reducing the burden on health systems and for investing money in favour of high priority health interventions [9,10]. Thus we were prompted to assess the proportion of interventions based on sound scientific base in a paediatric referral setting of a developing country.

## Methods

Referral care provided to children hospitalized in the paediatric department of the Instituto Especializado de Salud del Niño (IESN) was assessed for evidence base. IESN is a national paediatric hospital with more than 500 inpatient beds, serving mostly patients from deprived socioeconomic areas of Lima and inner cities of the country.

The clinical records of 195 children aged 1 month through 16 years old and hospitalized during January 2003 were initially revised.

One of the investigators (NYC) assessed the clinical records of children and assigned to each one a primary diagnosis and one or more primary therapeutic interventions, on the basis of the main clinical features and/or definitive diagnostic laboratory aids. Patients in whom a primary diagnosis was not possible to determine or those without a clear diagnosis were excluded.

The primary intervention was defined as the treatment or other manoeuvre that represented the most important attempt to cure, alleviate, or care for the patient in respect of his or her primary diagnosis [3].

To determine the level of evidence for each primary intervention, Cochrane reviews were searched. If there was not such a systematic review, a search through PubMed (MEDLINE) was performed by one of the investigators (NYC). All Cochrane reviews were searched through their own search tools. For PubMed, the period of search was 1966 through 2002. The key words used included those related to the primary diagnosis (e.g., pneumonia). Limits: "Title", "All child: 0–18 years". Publication type was sequentially searched for "Practice Guideline", "Meta-Analysis", "Randomized Controlled Trial" and "Review". Articles in English or Spanish were included. The National Guideline Clearinghouse was additionally visited for additional references. Published recommendations for judging the quality of guidelines were used for deciding the selection of the guidelines [11]. In addition, Clinical Evidence was used whenever deemed pertinent.

The level of evidence assigned to interventions was based on Ellis score (levels I, II and III) and the Oxford Centre for Evidence Based Medicine Levels of Evidence (grades A, B, C and D) [3,12]. Ellis score considers one or more interventions for a given diagnosis as one primary intervention. For comparison purposes we ranked each individual intervention through Oxford classification.

As a next step to our study, we planned the dissemination of the results among the hospital policy makers and the suggestion of corrective courses of action for those interventions needing improvement.

## Results

Results of the search strategies for PubMed are included as an appendix [See Additional File 1]. A guideline on management of pain in sickle cell disease was found in the National Guideline Clearinghouse website and the results of the search are also shown at the end of the appendix [See Additional File 1].

Overall, one hundred and ninety five clinical records were revised. Twenty eight clinical records were excluded because of undefined primary diagnosis (Table 1). One hundred and sixty seven remaining clinical records were further assessed. The most frequent primary diagnoses are shown in Table 2, being diarrhoeal dehydration and pneumonia the main causes for hospitalization. The childhood prevalent diseases are quite constant throughout the year at our hospital and thus it is unlikely that the results would have been different if we had chosen another study period.

Out of 167 primary interventions, 21% were supported by level I evidence, 73% were level II, and 6% were ranked as level III, according to Ellis classification [3]. Table 3 shows that most interventions classified as level I are referred to acute asthma exacerbations. Nebulized beta-agonists and systemic corticosteroids in bronchiolitis, and antibiotics for acute otitis media were considered level I according to Ellis. They were classified as D{5} according to Oxford Centre for Evidence Based Medicine Levels of Evidence (Table 3) [12].

Most assessed interventions were considered as level II (Table 4). Diarrhoeal dehydration and community-acquired pneumonia were the predominant diagnoses. Considering each prescription separately (fluid restriction, furosemide, spironolactone and captopril in heart failure, for example) we obtained 146 interventions. When we assessed them through the Oxford classification, 11% interventions were classified as grade B, 1% as grade C, and 88% as grade D. Appropriate interventions for the same diagnoses presented in Table 4 and ranked by Oxford classification are shown in Table 5.

Overtly unsubstantiated therapy according to Ellis classification is shown in Table 6. Considering levels I and II evidence-based therapy, 94% of therapeutic interventions were evidence based through Ellis classification. Using the Oxford classification, we obtained 193 individualized therapeutic interventions, that were classified as Grades A (16%), B (8%), C (1%), and D (75%).

Comparison of grade of recommendation of the prescribed intervention with the appropriate one is shown in Table 7. It will be used as summary evidence documenting our current hospital health care quality standard.

## Discussion

In this study 94% of therapeutic interventions were evidence-based by Ellis score. It may seem encouraging that more than 90% of therapeutic decisions in a referral paediatric hospital of a developing country are evidence based. However, the level II of evidence from Ellis includes interventions based in cohort studies, case-control studies, case series, expert's opinion, and even those based in basic sciences. We attempted therefore to classify the primary interventions according to more specific criteria. Using the Oxford classification, 75% of therapeutic interventions were based in expert opinions or in basic sciences (Grade of Recommendation D).

Some limitations of our study include the possible author' bias when assigning the primary diagnosis and primary intervention. The assignment of diagnosis by the clinician may have been influenced by both the choice of treatment and the available evidence. Only one of us classified the primary intervention. In addition, we evaluated only a primary intervention for a single primary diagnosis. Actually, many patients had more than one diagnosis and obviously more than one therapeutic intervention.

We used as evidence-base for rating the interventions assessed in our study, guidelines and evidence-based resources published in the developed world. This raises the issue of whether they are fully applicable to our setting. The most prevalent conditions found were diarrhoeal dehydration and community acquired pneumonia. For both of them we used British produced guidelines [20,21] because there were not Cochrane reviews on them and because the guidelines fulfilled recommended criteria for methodological quality of published guidelines [11]. The main recommendation of the guidelines on diarrhoea favours rapid oral rehydration over intravenous rehydration for children with mild to moderate dehydration [20]. This recommendation is based on several studies performed in both developed and developing countries and thus it can be applicable to both settings. The only concern on the applicability from setting to setting is that related to the osmolarity of the oral rehydration solution (ORS). The guidelines recommend a solution with 60 mmol/l of sodium, whereas a recent expert consensus found sufficient evidence to recommend the universal use of an ORS containing 75 mmol/l of sodium [49].

Regarding community acquired pneumonia, the British guidelines recommend antibiotic treatment for all children with pneumonia, due to the difficulties in identifying the aetiology, and they also specify criteria for hospitalization [21]. These recommendations are in agreement with the World Health Organization published guidelines [50]. The main difference is that the WHO guidelines rest on fast breathing and chest retraction for the diagnosis of pneumonia, whereas the British guidelines emphasize the role of chest x-rays. Chest x-rays are widely available in referral hospitals in developing countries and thus they should be used in addition to the clinical findings.

We acknowledge that the evidence derived from studies performed in developed countries should be translated with caution to developing settings. However, when the native research is scarce or of low quality, we think that the transfer of knowledge from the developed countries is an acceptable approach, as far as the particular characteristics of patients in developing countries are considered on an individual basis.

Dehydration due to diarrhoea and pneumonia were the most frequent diagnoses. Oral rehydration for diarrhoeal dehydration and antibiotics for pneumonia are considered as interventions with sufficient evidence for implementing them widely [9,10]. In our study, all children with mild to moderate dehydration were treated with slow intravenous infusion, and most children with uncomplicated community acquired pneumonia received intravenous antibiotics.

In addition to their enormous potential for saving lives, outpatient antibiotic therapy for pneumonia and outpatient oral rehydration can drastically reduce the rate of hospitalizations, the hospital stay, the hospital mortality rate, and the costs incurred. At our hospital, the mean stay time for hospitalized children is 4.7 days, and the mean crude mortality rate is 3.6%. We estimated the cost of managing hospitalized children with pneumonia and diarrhoeal dehydration as US$ 10.6/day and US$ 8.6/day, respectively. These costs are referred only to hospital bed and laboratory tests. A substantial amount of money could be saved treating these conditions on an outpatient basis.

We planned the dissemination of our results among the hospital policy makers. The tools that will be suggested for improving the standards include the development and systematic application of locally produced guidelines and/or the adaptation of published guidelines. A useful alternative that has been experienced for several years at our inpatient ward unit is to make available personal computers connected to Internet for attending physicians, residents and interns, and to encourage the use of online evidence-based resources. This last alternative may work better, particularly where there are motivated physicians who are able to lead the efforts for improving the health care standards. However, the ultimate decision to systematically introduce and monitor the suggested changes will rest on hospital managers. Such changes should also depend on taking into account the role of several other determinants of the clinical decision making by individual practitioners, such as continuous training, motivation, time, availability of drugs, equipments and supplies, supervision, and long-term health system strengthening strategies.

## Conclusions

Caution must be taken when assigning the level of evidence that supports therapeutic interventions, as commonly used classifications may be misleading. Existing effective interventions for dehydration and pneumonia are not being implemented in developing countries, even at referral level, and thus there is the need to change the current medical interventional behaviours. For the remaining diagnoses, the majority of assessed interventions were based on weak or non-existent evidence, highlighting the need to conduct appropriate clinical studies.

## Competing interests

The author(s) declare that they have no competing interests.

## Authors' contributions

NYC and LH conceived and designed the study. All authors analyzed and interpreted the data and contributed to earlier drafts of the manuscript.

## Pre-publication history

The pre-publication history for this paper can be accessed here:



## Supplementary Material

Additional File 1 "Appendix: Literature search results" details of the Medline (PubMed) search strategy and of the results are provided in this additional file. The selected articles are in black, bold characters. In addition, the search results from the National Guideline Clearinghouse website are provided for painful crisis in sickle cell anaemia. Click here for file

## Figures and Tables

**Table 1:** Patients excluded because of undefined diagnoses

**Diagnosis**	**N° of patients**
Convulsive syndrome	4
Acute obstructive bronchial syndrome	2
Acute rhinopharyngitis	2
Recurrent obstructive bronchial syndrome	2
Severe dehydration (referred from other hospitals)*	2
Acute lymphocytic leukaemia**	2
Unclassified dehydration	1
Emetic syndrome	1
Acute haemolytic anaemia of unknown cause	1
Anaemia of unknown cause	1
Thrombocytopenic purpura of unknown cause	1
Thrombocytopenia of unknown cause	1
Histiocytosis (clinical diagnosis)	1
Secondary epilepsy of unknown cause	1
Simple partial epilepsy of unknown cause	1
Munchausen's Syndrome (unclear criteria)	1
Congenital dyskeratosis	1
Neurocysticercosis (presumptive diagnosis)	1
Mental retardation (unspecified cause)	1
Operated hydrocephalousof unknown cause	1

**Total**	**28**

*Patients were fully hydrated upon arrival to IESN.

**Patients were referred to other hospital for definitive management.

**Table 2:** Definitive diagnoses

**Diagnosis**	**N° of patients**
Moderate diarrhoeal dehydration	48
Community acquired pneumonia	42
Acute asthma	22
Severe diarrhoeal dehydration	7
Congestive heart failure	6
Bronchiolitis	5
Mild diarrhoeal dehydration	4
Cellulitis	3
First simple febrile convulsion	3
Hypoxic crisis secondary to cyanotic congenital heart disease	3
Haemophilia A	3
Dilated cardiomyopathy	2
Nosocomial pneumonia	2
Post-infectious cerebellitis	1
Sickle cell anaemia vaso-occlusive crisis	1
Croup	1
Acute dysenteric diarrhoea	1
Invasive acute diarrhoea	1
Kawasaki disease	1
Anal fissure	1
Hydrocarbon poisoning	1
Recurrent urinary tract infection	1
Acute bacterial meningitis	1
Aspiration pneumonia	1
Acute otitis media	1
Mild acute pancreatitis	1
Immune thrombocytopenic purpura	1
Status epilepticus	1
Supraventricular tachycardia	1
Trichuriasis	1

**Total**	**167**

**Table 3:** Level I primary interventions (Ellis score) ranked by Oxford classification

**Diagnosis**	**Patients**	**Prescribed therapeutic intervention**	**Oxford classification**	**Appropriate therapeutic intervention**	**Oxford classification**	**Reference**
Acute asthma	22	Beta 2 agonists-Systemic corticosteroids	A{1a}	Beta 2 agonists-Systemic corticosteroids	A{1a}	13
Bronchiolitis	5	Beta 2 agonists-Systemic corticosteroids	D{5}	Supportive therapy	A{1a}	14
Simple febrile seizure	3	Observation	A{1a}	Observation	A{1a}	15
Cellulitis	2	Oxacillin	A{1b}	Oxacillin	A{1b}	16
Acute otitis media	1	Amoxicillin	D{5}	Antipyretic/analgesic therapy	A{1a}	17
Moderate croup	1	Dexamethasone L-Adrenaline	A{1a}, A{1b}	Dexamethasone L-Adrenaline	A{1a}, A{1b}	18
Trichuriasis	1	Albendazole – Mebendazole	A{1b}	Albendazole – Mebendazole	A{1b}	19

**Table 4:** Level II primary interventions (Ellis score) ranked by Oxford classification

**Diagnosis**	**Patients**	**Prescribed therapeutic intervention**	**Oxford classification**	**Reference**
Severe dehydration	1	IV ClNa 0.9% bolus, Rehydration in 04 hours	D{5}, B{2b}	20
Severe dehydration	6	IV ClNa 0.9% bolus, Rehydration in 24 hours	D{5}, D{5}	20
Moderate dehydration	1	IV 80 ml/Kg in 04 hours	B{2b}	20
Moderate dehydration	1	IV 80 ml/Kg in 06 hours	D{5}	20
Moderate dehydration	43	IV Rehydration in 24 hours	D{5}	20
Mild dehydration	4	IV Rehydration in 24 hours	D{5}	20
Severe dehydration	6	IV ClNa 0.9% bolus, Rehydration in 24 hours	D{5}, D5}	20
CA Pneumonia (<5 years)	33	Ampicillin/Ceftriaxone/Chloramphenicol	D{5}	21
CA Pneumonia (<5 years)	2	Ceftriaxone-Oxacillin/Ceftazidime-Vancomycin	D{5}	21
CA Pneumonia (<5 years)	1	Ampicillin-Amikacin	D{5}	21
CA Pneumonia (>5 years)	4	Penicillin G sodium	D{5}	21
CHF	2	Fluid restriction, Furosemide, Captopril, Spironolactone	D{5}, D{5}, B{2b}, B{1a}	22, 23, 24
CHF	3	Fluid restriction, Furosemide-Captopril	D{5}, D{5}, B{1a}	22, 23, 24
CHF	1	Fluid restriction, Hydrochlorothiazide, Captopril	D{5}, D{5}, B{1a}	22, 23, 24
Haemophilia A	3	Cryoprecipitate/Frozen fresh plasma	D{5}	25
Cyanotic crisis	2	Meperidine – Ethylephrine	D{5}	26
Nosocomial pneumonia	2	Ceftazidime-Vancomycin-Clindamycin	D{5}	27
Vaso-occlusive crisis (SCA)	1	Lysine-clonixinate	D{5}	28
Dilated cardiomyopathy	1	Lanatoside C-Furosemide-Captopril	D{5}, B{2b}	29, 30
Dilated cardiomyopathy	1	Furosemide-Captopril-Spironolactone	D{5}, B{2b}, D{5}	29, 30
Status epilepticus	1	Diazepam – Phenytoin	B{2a}	31, 32
Bacterial meningitis	1	Ceftriaxone	C	33
Dysenteric acute diarrhoea	1	Ceftriaxone	B{3b}	34
Invasive acute diarrhoea	1	Ceftriaxone	B{3b}	34
Recurrent UTI	1	Ciprofloxacin	C	35
Mild acute pancreatitis	1	Soft diet (low in fat) – Ranitidine	D{5}	36, 37, 38
Hydrocarbon ingestion	1	Nil per os – Soft diet	D{5}	39, 40
PSVT	1	Adenosine	B{1b}	41
Aspiration pneumonia	1	Chloramphenicol	D{5}	42
Post infectious cerebellitis	1	Acetaminophen	D5	43

CA: community acquired; CHF: congestive heart failure; SCA: sickle-cell anaemia; IV: intravenous; ORS: oral rehydration solution; PSVT: paroxysmal supraventricular tachycardia.

**Table 5:** Level II primary interventions (Ellis score) and their corresponding appropriate interventions ranked by Oxford classification*

**Diagnosis**	**Appropriate therapeutic intervention**	**Oxford classification**	**Reference**
Severe dehydration	IV 20 ml/Kg, 30–80 ml/Kg 3–4 hours	D{5}, B{2b}	20
Severe dehydration	IV 20 ml/Kg, 30–80 ml/Kg 3–4 hours	D{5}, B{2b}	20
Moderate dehydration	30–80 ml/Kg 3–4 hours (ORS)	B{2b}	20
Moderate dehydration	30–80 ml/Kg 3–4 hours (ORS)	B{2b}	20
Moderate dehydration	30–80 ml/Kg 3–4 hours (ORS)	B{2b}	20
Mild dehydration	30–50 ml/Kg 3–4 hours (ORS)	B{2b}	20
Severe dehydration	IV 20 ml/Kg, 30–80 ml/Kg 3–4 hours	D{5}, B{2b}	20
CA Pneumonia (<5 years)	Ampicillin/Chloramphenicol/Ceftriaxone	D{5}	21
CA Pneumonia (<5 years)	Ampicillin/Chloramphenicol/Ceftriaxone	D{5}	21
CA Pneumonia (<5 years)	Ampicillin/Chloramphenicol/Ceftriaxone	D{5}	21
CA Pneumonia (>5 years)	Penicillin G/Ceftriaxone/Cefuroxime	D{5}	21
CHF	Fluid restriction, Spironolactone, Captopril	D{5}, B{2b}, B{1a}	22, 23, 24
CHF	Fluid restriction, Spironolactone, Captopril	D{5}, B{2b}, B{1a}	22, 23, 24
CHF	Fluid restriction, Spironolactone, Captopril	D{5}, B{2b}, B{1a}	22, 23, 24
Haemophilia A	Factor VIII/Cryoprecipitate	D{5}	25
Cyanotic crisis	Meperidine	D{5}	26
Nosocomial pneumonia	Broad spectrum antibiotic therapy	D{5}	27
Vaso-occlusive crisis (SCA)	Ketorolac	C{2b}	28
Dilated cardiomyopathy	Digoxin-Furosemide, Captopril	D{5}, B{2b}	29, 30
Dilated cardiomyopathy	Digoxin-Furosemide, Captopril	D{5}, B2b}	29, 30
Status epilepticus	Diazepam – Phenytoin	B{2a}	31, 32
Bacterial meningitis	Ceftriaxone	C	33
Dysenteric acute diarrhoea	Ampicillin/Ceftriaxone	B{3b}	34
Invasive acute diarrhoea	Ampicillin/Ceftriaxone	B{3b}	34
Recurrent UTI	Empirical antibiotic therapy	C	35
Mild acute pancreatitis	Enteral feeding, Antibiotic, H2 Antagonists	D{5}	36, 37, 38
Hydrocarbon ingestion	Nil per os – Low fat diet	D{5}	39, 40
PSVT	Adenosine	B{1b}	41
Aspiration pneumonia	Penicillin/Clindamycin	A{1b}	42
Post infectious cerebellitis	Intravenous immunoglobulin	C4	43

*Diagnoses are the same as in Table 4. CA: community acquired; CHF: congestive heart failure; SCA: sickle-cell anaemia; IV: intravenous; ORS: oral rehydration solution; PSVT: paroxysmal supraventricular tachycardia.

**Table 6:** Level III primary interventions (Ellis score) ranked by Oxford classification

**Diagnosis**	**Patients**	**Prescribed therapeutic intervention**	**Oxford classification**	**Appropriate therapeutical intervention**	**Oxford classification**	**Reference**
Moderate dehydration	3	IV 0.9% ClNa bolus – Time of rehydration: 24 hours	D	Oral rehydration solution, 30–80 cc/Kg in 3–4 hours	B{2b}	20
Community acquired pneumonia (<5 years)	2	Antibiotic therapy <3 days	D	Ampicillin/Chloramphenicol/Ceftriaxone	D{5}	21
Cyanotic crisis due to cyanotic congenital heart disease	1	Meperidine, Ethylephrine, Oxygen	D{5}, D	Meperidine	D{5}	26
Facial cellulitis	1	Ceftazidime, Vancomycin	D{5}	Oxacillin, Cephalosporin	D{5}	20
Idiopathic thrombocytopenic purpura*	1	Platelets, Dexamethasone	D{5}	Systemic corticosteroids	A{1b}	44
Anal fissure	1	Zinc oxide	D	Topical anaesthetic or glyceryl trinitrate	A{1b}	45, 46
Kawasaki disease	1	Acetylsalicylic acid	D	Intravenous immunoglobulin, acetylsalicylic acid	A{1a}	47, 48

*Presenting as haematoma with 15,000 plateles/mm^3^

**Table 7:** Therapeutic interventions graded according to Oxford classification

	**Prescribed intervention**		**Appropriate intervention**	
	
**Grade of recommendation**	**N°**	**%**	**N°**	**%**
				
**A**	30	16	38	20
**B**	16	8	77	41
**C**	2	1	4	2
**D**	145	75	70	37
**Total**	193	100	189	100
