# Supplementary material for: Audit of therapeutic interventions in inpatient children using two scores: are they evidence-based in developing countries?
Source: BMC Health Serv Res. 2004 Dec 29;4:40. doi: 10.1186/1472-6963-4-40 (PMC544399; doi:10.1186/1472-6963-4-40)
Supplement: Additional File 1 — "Appendix: Literature search results" details of the Medline (PubMed) search strategy and of the results are provided in this additional file. The selected articles are in black, bold characters. In addition, the search results from the National Guideline Clearinghouse website are provided for painful crisis in sickle cell anaemia. [file 1472-6963-4-40-S1.doc]

**Appendix: Literature search results**

- Febrile seizure:

| | [Entrez](http://www.ncbi.nlm.nih.gov/gquery/gquery.fcgi?itool=toolbar) | [PubMed](http://www.ncbi.nlm.nih.gov/entrez/query.fcgi?db=PubMed&itool=toolbar) | [Nucleotide](http://www.ncbi.nlm.nih.gov/entrez/query.fcgi?db=Nucleotide&itool=toolbar) | [Protein](http://www.ncbi.nlm.nih.gov/entrez/query.fcgi?db=Protein&itool=toolbar) | [Genome](http://www.ncbi.nlm.nih.gov/entrez/query.fcgi?db=Genome&itool=toolbar) | [Structure](http://www.ncbi.nlm.nih.gov/entrez/query.fcgi?db=Structure&itool=toolbar) | [OMIM](http://www.ncbi.nlm.nih.gov/entrez/query.fcgi?db=OMIM&itool=toolbar) | [PMC](http://www.ncbi.nlm.nih.gov/entrez/query.fcgi?db=PMC&itool=toolbar) | [Journals](http://www.ncbi.nlm.nih.gov/entrez/query.fcgi?db=Journals&itool=toolbar) | [Books](http://www.ncbi.nlm.nih.gov/entrez/query.fcgi?db=Books&itool=toolbar) | | --- | --- | --- | --- | --- | --- | --- | --- | --- | --- | |
| --- | --- | --- | --- | --- | --- | --- | --- | --- | --- | --- |
| | Search for | | --- | |
| |  | |  | [Limits](javascript:Go('Limits')) | [Preview/Index](javascript:Go('Index')) | [History](javascript:Go('History')) | [Clipboard](javascript:Go('Clipboard')) | [Details](javascript:Go('Details')) |  |  | | --- | --- | --- | --- | --- | --- | --- | --- | | | --- | --- | --- | --- | --- | --- | --- | --- | --- | --- | |

|  | | |
| --- | --- | --- |
| [About Entrez](http://www.ncbi.nlm.nih.gov/Database/index.html)  [Text Version](http://www.ncbi.nlm.nih.gov/entrez/queryd.fcgi?linkbar=plain)  Entrez PubMed  [Overview](http://www.ncbi.nlm.nih.gov/entrez/query/static/overview.html) [Help |](http://www.ncbi.nlm.nih.gov/entrez/query/static/help/pmhelp.html) [FAQ](http://www.ncbi.nlm.nih.gov/entrez/query/static/faq.html) [Tutorial](http://www.nlm.nih.gov/bsd/pubmed_tutorial/m1001.html) [New/Noteworthy](http://www.ncbi.nlm.nih.gov/entrez/query/static/new.html) [E-Utilities](http://eutils.ncbi.nlm.nih.gov/entrez/query/static/eutils_help.html)  PubMed Services [Journals Database](http://www.ncbi.nlm.nih.gov/entrez/query.fcgi?db=journals) [MeSH Database](http://www.ncbi.nlm.nih.gov/entrez/query.fcgi?db=mesh) [Single Citation Matcher](http://www.ncbi.nlm.nih.gov/entrez/query/static/citmatch.html) [Batch Citation Matcher](http://www.ncbi.nlm.nih.gov/entrez/getids.cgi) [Clinical Queries](http://www.ncbi.nlm.nih.gov/entrez/query/static/clinical.html) [LinkOut](http://www.ncbi.nlm.nih.gov/entrez/linkout) [Cubby](http://www.ncbi.nlm.nih.gov/entrez/cubby.fcgi?call=QueryExt.Query.last.Show&call=QueryExt.CubbyQuery..ShowAll)  Related Resources [Order Documents](http://www.nlm.nih.gov/loansomedoc/loansome_home.html) [NLM Catalog](http://www.ncbi.nlm.nih.gov/entrez/query.fcgi?db=nlmcatalog) [NLM Gateway](http://gateway.nlm.nih.gov/gw/Cmd) [TOXNET](http://toxnet.nlm.nih.gov/) [Consumer Health](http://www.nlm.nih.gov/medlineplus/) [Clinical Alerts](http://www.nlm.nih.gov/databases/alerts/clinical_alerts.html) [ClinicalTrials.gov](http://clinicaltrials.gov/ct/gui) [PubMed Central](http://www.pubmedcentral.nih.gov/) |  | | Field: **Title,** Limits: **All Child: 0-18 years, Publication Date to 2002, Practice Guideline** | | --- |  | Show: | | --- | |  |  | **1:** | [**[No authors listed]**](http://www.ncbi.nlm.nih.gov/entrez/query.fcgi?cmd=Retrieve&db=pubmed&dopt=Abstract&list_uids=8628629) | [Related Articles,](http://www.ncbi.nlm.nih.gov/entrez/query.fcgi?db=pubmed&cmd=Display&dopt=pubmed_pubmed&from_uid=8628629) 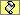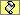[Links](javascript:PopUpMenu2_Set(Menu8628629);) | | --- | --- | --- | | [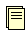](http://www.ncbi.nlm.nih.gov/entrez/query.fcgi?cmd=Retrieve&db=pubmed&dopt=Abstract&list_uids=8628629&itool=iconabstr) | **Practice parameter: the neurodiagnostic evaluation of the child with a first simple febrile seizure. American Academy of Pediatrics. Provisional Committee on Quality Improvement, Subcommittee on Febrile Seizures. Pediatrics. 1996 May;97(5):769-72; discussion 773-5.** PMID: 8628629 [PubMed - indexed for MEDLINE] | | |

- Skin infection:

| | [Entrez](http://www.ncbi.nlm.nih.gov/gquery/gquery.fcgi?itool=toolbar) | [PubMed](http://www.ncbi.nlm.nih.gov/entrez/query.fcgi?db=PubMed&itool=toolbar) | [Nucleotide](http://www.ncbi.nlm.nih.gov/entrez/query.fcgi?db=Nucleotide&itool=toolbar) | [Protein](http://www.ncbi.nlm.nih.gov/entrez/query.fcgi?db=Protein&itool=toolbar) | [Genome](http://www.ncbi.nlm.nih.gov/entrez/query.fcgi?db=Genome&itool=toolbar) | [Structure](http://www.ncbi.nlm.nih.gov/entrez/query.fcgi?db=Structure&itool=toolbar) | [OMIM](http://www.ncbi.nlm.nih.gov/entrez/query.fcgi?db=OMIM&itool=toolbar) | [PMC](http://www.ncbi.nlm.nih.gov/entrez/query.fcgi?db=PMC&itool=toolbar) | [Journals](http://www.ncbi.nlm.nih.gov/entrez/query.fcgi?db=Journals&itool=toolbar) | [Books](http://www.ncbi.nlm.nih.gov/entrez/query.fcgi?db=Books&itool=toolbar) | | --- | --- | --- | --- | --- | --- | --- | --- | --- | --- | |
| --- | --- | --- | --- | --- | --- | --- | --- | --- | --- | --- |
| | Search for | | --- | |
| |  | |  | [Limits](javascript:Go('Limits')) | [Preview/Index](javascript:Go('Index')) | [History](javascript:Go('History')) | [Clipboard](javascript:Go('Clipboard')) | [Details](javascript:Go('Details')) |  |  | | --- | --- | --- | --- | --- | --- | --- | --- | | | --- | --- | --- | --- | --- | --- | --- | --- | --- | --- | |

|  | | |
| --- | --- | --- |
| [About Entrez](http://www.ncbi.nlm.nih.gov/Database/index.html)  [Text Version](http://www.ncbi.nlm.nih.gov/entrez/queryd.fcgi?linkbar=plain)  Entrez PubMed  [Overview](http://www.ncbi.nlm.nih.gov/entrez/query/static/overview.html) [Help |](http://www.ncbi.nlm.nih.gov/entrez/query/static/help/pmhelp.html) [FAQ](http://www.ncbi.nlm.nih.gov/entrez/query/static/faq.html) [Tutorial](http://www.nlm.nih.gov/bsd/pubmed_tutorial/m1001.html) [New/Noteworthy](http://www.ncbi.nlm.nih.gov/entrez/query/static/new.html) [E-Utilities](http://eutils.ncbi.nlm.nih.gov/entrez/query/static/eutils_help.html)  PubMed Services [Journals Database](http://www.ncbi.nlm.nih.gov/entrez/query.fcgi?db=journals) [MeSH Database](http://www.ncbi.nlm.nih.gov/entrez/query.fcgi?db=mesh) [Single Citation Matcher](http://www.ncbi.nlm.nih.gov/entrez/query/static/citmatch.html) [Batch Citation Matcher](http://www.ncbi.nlm.nih.gov/entrez/getids.cgi) [Clinical Queries](http://www.ncbi.nlm.nih.gov/entrez/query/static/clinical.html) [LinkOut](http://www.ncbi.nlm.nih.gov/entrez/linkout) [Cubby](http://www.ncbi.nlm.nih.gov/entrez/cubby.fcgi?call=QueryExt.Query.last.Show&call=QueryExt.CubbyQuery..ShowAll)  Related Resources [Order Documents](http://www.nlm.nih.gov/loansomedoc/loansome_home.html) [NLM Catalog](http://www.ncbi.nlm.nih.gov/entrez/query.fcgi?db=nlmcatalog) [NLM Gateway](http://gateway.nlm.nih.gov/gw/Cmd) [TOXNET](http://toxnet.nlm.nih.gov/) [Consumer Health](http://www.nlm.nih.gov/medlineplus/) [Clinical Alerts](http://www.nlm.nih.gov/databases/alerts/clinical_alerts.html) [ClinicalTrials.gov](http://clinicaltrials.gov/ct/gui) [PubMed Central](http://www.pubmedcentral.nih.gov/) |  | | Field: **Title,** Limits: **Publication Date to 2002, Randomized Controlled Trial** | | --- |  | Show: | | --- | |  |  | Items 1 - 2 of 2 | One page. | | --- | --- |  | **1:** | [**Stevens DL, Smith LG, Bruss JB, McConnell-Martin MA, Duvall SE, Todd WM, Hafkin B.**](http://www.ncbi.nlm.nih.gov/entrez/query.fcgi?cmd=Retrieve&db=pubmed&dopt=Abstract&list_uids=11083648) | [Related Articles,](http://www.ncbi.nlm.nih.gov/entrez/query.fcgi?db=pubmed&cmd=Display&dopt=pubmed_pubmed&from_uid=11083648) 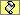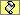[Links](javascript:PopUpMenu2_Set(Menu11083648);) | | --- | --- | --- | | [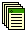](http://www.ncbi.nlm.nih.gov/entrez/query.fcgi?cmd=Retrieve&db=pubmed&dopt=Abstract&list_uids=11083648&itool=iconfft) | **Randomized comparison of linezolid (PNU-100766) versus oxacillin-dicloxacillin for treatment of complicated skin and soft tissue infections. Antimicrob Agents Chemother. 2000 Dec;44(12):3408-13.** PMID: 11083648 [PubMed - indexed for MEDLINE] | |  | **2:** | [Nichols RL, Graham DR, Barriere SL, Rodgers A, Wilson SE, Zervos M, Dunn DL, Kreter B.](http://www.ncbi.nlm.nih.gov/entrez/query.fcgi?cmd=Retrieve&db=pubmed&dopt=Abstract&list_uids=10473234) | [Related Articles,](http://www.ncbi.nlm.nih.gov/entrez/query.fcgi?db=pubmed&cmd=Display&dopt=pubmed_pubmed&from_uid=10473234) 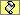[Links](javascript:PopUpMenu2_Set(Menu10473234);) | | --- | --- | --- | | [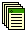](http://www.ncbi.nlm.nih.gov/entrez/query.fcgi?cmd=Retrieve&db=pubmed&dopt=Abstract&list_uids=10473234&itool=iconfft) | Treatment of hospitalized patients with complicated gram-positive skin and skin structure infections: two randomized, multicentre studies of quinupristin/dalfopristin versus cefazolin, oxacillin or vancomycin. Synercid Skin and Skin Structure Infection Group. J Antimicrob Chemother. 1999 Aug;44(2):263-73.Erratum in: J Antimicrob Chemother 1999 Oct;44(4):585.  PMID: 10473234 [PubMed - indexed for MEDLINE] | | |

- Trichuriasis:

| | [Entrez](http://www.ncbi.nlm.nih.gov/gquery/gquery.fcgi?itool=toolbar) | [PubMed](http://www.ncbi.nlm.nih.gov/entrez/query.fcgi?db=PubMed&itool=toolbar) | [Nucleotide](http://www.ncbi.nlm.nih.gov/entrez/query.fcgi?db=Nucleotide&itool=toolbar) | [Protein](http://www.ncbi.nlm.nih.gov/entrez/query.fcgi?db=Protein&itool=toolbar) | [Genome](http://www.ncbi.nlm.nih.gov/entrez/query.fcgi?db=Genome&itool=toolbar) | [Structure](http://www.ncbi.nlm.nih.gov/entrez/query.fcgi?db=Structure&itool=toolbar) | [OMIM](http://www.ncbi.nlm.nih.gov/entrez/query.fcgi?db=OMIM&itool=toolbar) | [PMC](http://www.ncbi.nlm.nih.gov/entrez/query.fcgi?db=PMC&itool=toolbar) | [Journals](http://www.ncbi.nlm.nih.gov/entrez/query.fcgi?db=Journals&itool=toolbar) | [Books](http://www.ncbi.nlm.nih.gov/entrez/query.fcgi?db=Books&itool=toolbar) | | --- | --- | --- | --- | --- | --- | --- | --- | --- | --- | |
| --- | --- | --- | --- | --- | --- | --- | --- | --- | --- | --- |
| | Search for | | --- | |
| |  | |  | [Limits](javascript:Go('Limits')) | [Preview/Index](javascript:Go('Index')) | [History](javascript:Go('History')) | [Clipboard](javascript:Go('Clipboard')) | [Details](javascript:Go('Details')) |  |  | | --- | --- | --- | --- | --- | --- | --- | --- | | | --- | --- | --- | --- | --- | --- | --- | --- | --- | --- | |

|  | | |
| --- | --- | --- |
| [About Entrez](http://www.ncbi.nlm.nih.gov/Database/index.html)  [Text Version](http://www.ncbi.nlm.nih.gov/entrez/queryd.fcgi?linkbar=plain)  Entrez PubMed  [Overview](http://www.ncbi.nlm.nih.gov/entrez/query/static/overview.html) [Help |](http://www.ncbi.nlm.nih.gov/entrez/query/static/help/pmhelp.html) [FAQ](http://www.ncbi.nlm.nih.gov/entrez/query/static/faq.html) [Tutorial](http://www.nlm.nih.gov/bsd/pubmed_tutorial/m1001.html) [New/Noteworthy](http://www.ncbi.nlm.nih.gov/entrez/query/static/new.html) [E-Utilities](http://eutils.ncbi.nlm.nih.gov/entrez/query/static/eutils_help.html)  PubMed Services [Journals Database](http://www.ncbi.nlm.nih.gov/entrez/query.fcgi?db=journals) [MeSH Database](http://www.ncbi.nlm.nih.gov/entrez/query.fcgi?db=mesh) [Single Citation Matcher](http://www.ncbi.nlm.nih.gov/entrez/query/static/citmatch.html) [Batch Citation Matcher](http://www.ncbi.nlm.nih.gov/entrez/getids.cgi) [Clinical Queries](http://www.ncbi.nlm.nih.gov/entrez/query/static/clinical.html) [LinkOut](http://www.ncbi.nlm.nih.gov/entrez/linkout) [Cubby](http://www.ncbi.nlm.nih.gov/entrez/cubby.fcgi?call=QueryExt.Query.last.Show&call=QueryExt.CubbyQuery..ShowAll)  Related Resources [Order Documents](http://www.nlm.nih.gov/loansomedoc/loansome_home.html) [NLM Catalog](http://www.ncbi.nlm.nih.gov/entrez/query.fcgi?db=nlmcatalog) [NLM Gateway](http://gateway.nlm.nih.gov/gw/Cmd) [TOXNET](http://toxnet.nlm.nih.gov/) [Consumer Health](http://www.nlm.nih.gov/medlineplus/) [Clinical Alerts](http://www.nlm.nih.gov/databases/alerts/clinical_alerts.html) [ClinicalTrials.gov](http://clinicaltrials.gov/ct/gui) [PubMed Central](http://www.pubmedcentral.nih.gov/) |  | | Field: **Title,** Limits: **All Child: 0-18 years, Publication Date to 2002, Randomized Controlled Trial** | | --- |  | Show: | | --- | | **** |  | **1:** | [**Jongsuksuntigul P, Jeradit C, Pornpattanakul S, Charanasri U.**](http://www.ncbi.nlm.nih.gov/entrez/query.fcgi?cmd=Retrieve&db=pubmed&dopt=Abstract&list_uids=7939948) | [Related Articles,](http://www.ncbi.nlm.nih.gov/entrez/query.fcgi?db=pubmed&cmd=Display&dopt=pubmed_pubmed&from_uid=7939948) 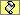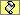[Links](javascript:PopUpMenu2_Set(Menu7939948);) | | --- | --- | --- | | **[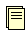](http://www.ncbi.nlm.nih.gov/entrez/query.fcgi?cmd=Retrieve&db=pubmed&dopt=Abstract&list_uids=7939948&itool=iconabstr)** | **A comparative study on the efficacy of albendazole and mebendazole in the treatment of ascariasis, hookworm infection and trichuriasis. Southeast Asian J Trop Med Public Health. 1993 Dec;24(4):724-9. PMID: 7939948 [PubMed - indexed for MEDLINE]** | | |

- Diarrhoeal dehydration:

| | [Entrez](http://www.ncbi.nlm.nih.gov/gquery/gquery.fcgi?itool=toolbar) | [PubMed](http://www.ncbi.nlm.nih.gov/entrez/query.fcgi?db=PubMed&itool=toolbar) | [Nucleotide](http://www.ncbi.nlm.nih.gov/entrez/query.fcgi?db=Nucleotide&itool=toolbar) | [Protein](http://www.ncbi.nlm.nih.gov/entrez/query.fcgi?db=Protein&itool=toolbar) | [Genome](http://www.ncbi.nlm.nih.gov/entrez/query.fcgi?db=Genome&itool=toolbar) | [Structure](http://www.ncbi.nlm.nih.gov/entrez/query.fcgi?db=Structure&itool=toolbar) | [OMIM](http://www.ncbi.nlm.nih.gov/entrez/query.fcgi?db=OMIM&itool=toolbar) | [PMC](http://www.ncbi.nlm.nih.gov/entrez/query.fcgi?db=PMC&itool=toolbar) | [Journals](http://www.ncbi.nlm.nih.gov/entrez/query.fcgi?db=Journals&itool=toolbar) | [Books](http://www.ncbi.nlm.nih.gov/entrez/query.fcgi?db=Books&itool=toolbar) | | --- | --- | --- | --- | --- | --- | --- | --- | --- | --- | |
| --- | --- | --- | --- | --- | --- | --- | --- | --- | --- | --- |
| | Search for | | --- | |
| |  | |  | [Limits](javascript:Go('Limits')) | [Preview/Index](javascript:Go('Index')) | [History](javascript:Go('History')) | [Clipboard](javascript:Go('Clipboard')) | [Details](javascript:Go('Details')) |  |  | | --- | --- | --- | --- | --- | --- | --- | --- | | | --- | --- | --- | --- | --- | --- | --- | --- | --- | --- | |

|  | | |
| --- | --- | --- |
| [About Entrez](http://www.ncbi.nlm.nih.gov/Database/index.html)  [Text Version](http://www.ncbi.nlm.nih.gov/entrez/queryd.fcgi?linkbar=plain)  Entrez PubMed  [Overview](http://www.ncbi.nlm.nih.gov/entrez/query/static/overview.html) [Help |](http://www.ncbi.nlm.nih.gov/entrez/query/static/help/pmhelp.html) [FAQ](http://www.ncbi.nlm.nih.gov/entrez/query/static/faq.html) [Tutorial](http://www.nlm.nih.gov/bsd/pubmed_tutorial/m1001.html) [New/Noteworthy](http://www.ncbi.nlm.nih.gov/entrez/query/static/new.html) [E-Utilities](http://eutils.ncbi.nlm.nih.gov/entrez/query/static/eutils_help.html)  PubMed Services [Journals Database](http://www.ncbi.nlm.nih.gov/entrez/query.fcgi?db=journals) [MeSH Database](http://www.ncbi.nlm.nih.gov/entrez/query.fcgi?db=mesh) [Single Citation Matcher](http://www.ncbi.nlm.nih.gov/entrez/query/static/citmatch.html) [Batch Citation Matcher](http://www.ncbi.nlm.nih.gov/entrez/getids.cgi) [Clinical Queries](http://www.ncbi.nlm.nih.gov/entrez/query/static/clinical.html) [LinkOut](http://www.ncbi.nlm.nih.gov/entrez/linkout) [Cubby](http://www.ncbi.nlm.nih.gov/entrez/cubby.fcgi?call=QueryExt.Query.last.Show&call=QueryExt.CubbyQuery..ShowAll)  Related Resources [Order Documents](http://www.nlm.nih.gov/loansomedoc/loansome_home.html) [NLM Catalog](http://www.ncbi.nlm.nih.gov/entrez/query.fcgi?db=nlmcatalog) [NLM Gateway](http://gateway.nlm.nih.gov/gw/Cmd) [TOXNET](http://toxnet.nlm.nih.gov/) [Consumer Health](http://www.nlm.nih.gov/medlineplus/) [Clinical Alerts](http://www.nlm.nih.gov/databases/alerts/clinical_alerts.html) [ClinicalTrials.gov](http://clinicaltrials.gov/ct/gui) [PubMed Central](http://www.pubmedcentral.nih.gov/) |  | | Field: **Title,** Limits: **All Child: 0-18 years, Publication Date to 2002, Practice Guideline** | | --- |  | Show: | | --- | |  |  | Items 1 - 3 of 3 | One page. | | --- | --- |  | **1:** | [Guarino A, Albano F; Working Group on Intestinal Infections of the Italian Society of Paediatric Gastroenterology and Hepatology.](http://www.ncbi.nlm.nih.gov/entrez/query.fcgi?cmd=Retrieve&db=pubmed&dopt=Abstract&list_uids=11697412) | [Related Articles,](http://www.ncbi.nlm.nih.gov/entrez/query.fcgi?db=pubmed&cmd=Display&dopt=pubmed_pubmed&from_uid=11697412) 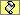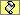[Links](javascript:PopUpMenu2_Set(Menu11697412);) | | --- | --- | --- | | [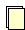](http://www.ncbi.nlm.nih.gov/entrez/query.fcgi?cmd=Retrieve&db=pubmed&dopt=Abstract&list_uids=11697412&itool=iconnoabstr) | Guidelines for the approach to outpatient children with acute diarrhoea. Acta Paediatr. 2001 Oct;90(10):1087-95. No abstract available. PMID: 11697412 [PubMed - indexed for MEDLINE] | |  | **2:** | [**Armon K, Stephenson T, MacFaul R, Eccleston P, Werneke U.**](http://www.ncbi.nlm.nih.gov/entrez/query.fcgi?cmd=Retrieve&db=pubmed&dopt=Abstract&list_uids=11466188) | [Related Articles,](http://www.ncbi.nlm.nih.gov/entrez/query.fcgi?db=pubmed&cmd=Display&dopt=pubmed_pubmed&from_uid=11466188) 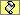[Links](javascript:PopUpMenu2_Set(Menu11466188);) | | --- | --- | --- | | [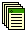](http://www.ncbi.nlm.nih.gov/entrez/query.fcgi?cmd=Retrieve&db=pubmed&dopt=Abstract&list_uids=11466188&itool=iconfft) | **An evidence and consensus based guideline for acute diarrhoea management. Arch Dis Child. 2001 Aug;85(2):132-42. Review.** PMID: 11466188 [PubMed - indexed for MEDLINE] | |  | **3:** | [Walker-Smith JA, Sandhu BK, Isolauri E, Banchini G, van Caillie-Bertrand M, Dias JA, Fasano A, Guandalini S, Hoekstra JH, Juntunen M, Kolacek S, Marx D, Micetic-Turk D, Razenberg MC, Szajewska H, Taminiau J, Weizman Z, Zanacca C, Zetterstrom R.](http://www.ncbi.nlm.nih.gov/entrez/query.fcgi?cmd=Retrieve&db=pubmed&dopt=Abstract&list_uids=9161963) | [Related Articles,](http://www.ncbi.nlm.nih.gov/entrez/query.fcgi?db=pubmed&cmd=Display&dopt=pubmed_pubmed&from_uid=9161963) 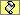[Links](javascript:PopUpMenu2_Set(Menu9161963);) | | --- | --- | --- | | [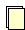](http://www.ncbi.nlm.nih.gov/entrez/query.fcgi?cmd=Retrieve&db=pubmed&dopt=Abstract&list_uids=9161963&itool=iconnoabstr) | Guidelines prepared by the ESPGAN Working Group on Acute Diarrhoea. Recommendations for feeding in childhood gastroenteritis. European Society of Pediatric Gastroenterology and Nutrition. J Pediatr Gastroenterol Nutr. 1997 May;24(5):619-20. No abstract available. PMID: 9161963 [PubMed - indexed for MEDLINE] | | |

- Pneumonia:

| | [Entrez](http://www.ncbi.nlm.nih.gov/gquery/gquery.fcgi?itool=toolbar) | [PubMed](http://www.ncbi.nlm.nih.gov/entrez/query.fcgi?db=PubMed&itool=toolbar) | [Nucleotide](http://www.ncbi.nlm.nih.gov/entrez/query.fcgi?db=Nucleotide&itool=toolbar) | [Protein](http://www.ncbi.nlm.nih.gov/entrez/query.fcgi?db=Protein&itool=toolbar) | [Genome](http://www.ncbi.nlm.nih.gov/entrez/query.fcgi?db=Genome&itool=toolbar) | [Structure](http://www.ncbi.nlm.nih.gov/entrez/query.fcgi?db=Structure&itool=toolbar) | [OMIM](http://www.ncbi.nlm.nih.gov/entrez/query.fcgi?db=OMIM&itool=toolbar) | [PMC](http://www.ncbi.nlm.nih.gov/entrez/query.fcgi?db=PMC&itool=toolbar) | [Journals](http://www.ncbi.nlm.nih.gov/entrez/query.fcgi?db=Journals&itool=toolbar) | [Books](http://www.ncbi.nlm.nih.gov/entrez/query.fcgi?db=Books&itool=toolbar) | | --- | --- | --- | --- | --- | --- | --- | --- | --- | --- | |
| --- | --- | --- | --- | --- | --- | --- | --- | --- | --- | --- |
| | Search for | | --- | |
| |  | |  | [Limits](javascript:Go('Limits')) | [Preview/Index](javascript:Go('Index')) | [History](javascript:Go('History')) | [Clipboard](javascript:Go('Clipboard')) | [Details](javascript:Go('Details')) |  |  | | --- | --- | --- | --- | --- | --- | --- | --- | | | --- | --- | --- | --- | --- | --- | --- | --- | --- | --- | |

|  | | |
| --- | --- | --- |
| [About Entrez](http://www.ncbi.nlm.nih.gov/Database/index.html)  [Text Version](http://www.ncbi.nlm.nih.gov/entrez/queryd.fcgi?linkbar=plain)  Entrez PubMed  [Overview](http://www.ncbi.nlm.nih.gov/entrez/query/static/overview.html) [Help |](http://www.ncbi.nlm.nih.gov/entrez/query/static/help/pmhelp.html) [FAQ](http://www.ncbi.nlm.nih.gov/entrez/query/static/faq.html) [Tutorial](http://www.nlm.nih.gov/bsd/pubmed_tutorial/m1001.html) [New/Noteworthy](http://www.ncbi.nlm.nih.gov/entrez/query/static/new.html) [E-Utilities](http://eutils.ncbi.nlm.nih.gov/entrez/query/static/eutils_help.html)  PubMed Services [Journals Database](http://www.ncbi.nlm.nih.gov/entrez/query.fcgi?db=journals) [MeSH Database](http://www.ncbi.nlm.nih.gov/entrez/query.fcgi?db=mesh) [Single Citation Matcher](http://www.ncbi.nlm.nih.gov/entrez/query/static/citmatch.html) [Batch Citation Matcher](http://www.ncbi.nlm.nih.gov/entrez/getids.cgi) [Clinical Queries](http://www.ncbi.nlm.nih.gov/entrez/query/static/clinical.html) [LinkOut](http://www.ncbi.nlm.nih.gov/entrez/linkout) [Cubby](http://www.ncbi.nlm.nih.gov/entrez/cubby.fcgi?call=QueryExt.Query.last.Show&call=QueryExt.CubbyQuery..ShowAll)  Related Resources [Order Documents](http://www.nlm.nih.gov/loansomedoc/loansome_home.html) [NLM Catalog](http://www.ncbi.nlm.nih.gov/entrez/query.fcgi?db=nlmcatalog) [NLM Gateway](http://gateway.nlm.nih.gov/gw/Cmd) [TOXNET](http://toxnet.nlm.nih.gov/) [Consumer Health](http://www.nlm.nih.gov/medlineplus/) [Clinical Alerts](http://www.nlm.nih.gov/databases/alerts/clinical_alerts.html) [ClinicalTrials.gov](http://clinicaltrials.gov/ct/gui) [PubMed Central](http://www.pubmedcentral.nih.gov/) |  | | Field: **Title,** Limits: **All Child: 0-18 years, Publication Date to 2002, Practice Guideline** | | --- |  | Show: | | --- | |  |  | Items 1 - 12 of 12 | One page. | | --- | --- |  | **1:** | [**British Thoracic Society Standards of Care Committee.**](http://www.ncbi.nlm.nih.gov/entrez/query.fcgi?cmd=Retrieve&db=pubmed&dopt=Abstract&list_uids=11994552) | [Related Articles,](http://www.ncbi.nlm.nih.gov/entrez/query.fcgi?db=pubmed&cmd=Display&dopt=pubmed_pubmed&from_uid=11994552) 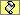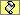[Links](javascript:PopUpMenu2_Set(Menu11994552);) | | --- | --- | --- | | [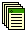](http://www.ncbi.nlm.nih.gov/entrez/query.fcgi?cmd=Retrieve&db=pubmed&dopt=Abstract&list_uids=11994552&itool=iconfft) | **British Thoracic Society Guidelines for the Management of Community Acquired Pneumonia in Childhood. Thorax. 2002 May;57 Suppl 1:i1-24. No abstract available.** PMID: 11994552 [PubMed - indexed for MEDLINE] | |  | **2:** | [American College of Emergency Physicians.](http://www.ncbi.nlm.nih.gov/entrez/query.fcgi?cmd=Retrieve&db=pubmed&dopt=Abstract&list_uids=11859897) | [Related Articles,](http://www.ncbi.nlm.nih.gov/entrez/query.fcgi?db=pubmed&cmd=Display&dopt=pubmed_pubmed&from_uid=11859897) 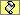[Links](javascript:PopUpMenu2_Set(Menu11859897);) | | --- | --- | --- | | [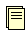](http://www.ncbi.nlm.nih.gov/entrez/query.fcgi?cmd=Retrieve&db=pubmed&dopt=Abstract&list_uids=11859897&itool=iconabstr) | Clinical policy for the management and risk stratification of community-acquired pneumonia in adults in the emergency department. Ann Emerg Med. 2001 Jul;38(1):107-13. PMID: 11859897 [PubMed - indexed for MEDLINE] | |  | **3:** | [Mandell LA, Marrie TJ, Grossman RF, Chow AW, Hyland RH.](http://www.ncbi.nlm.nih.gov/entrez/query.fcgi?cmd=Retrieve&db=pubmed&dopt=Abstract&list_uids=10987698) | [Related Articles,](http://www.ncbi.nlm.nih.gov/entrez/query.fcgi?db=pubmed&cmd=Display&dopt=pubmed_pubmed&from_uid=10987698) 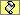[Links](javascript:PopUpMenu2_Set(Menu10987698);) | | --- | --- | --- | | [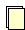](http://www.ncbi.nlm.nih.gov/entrez/query.fcgi?cmd=Retrieve&db=pubmed&dopt=Abstract&list_uids=10987698&itool=iconnoabstr) | Canadian guidelines for the initial management of community-acquired pneumonia: an evidence-based update by the Canadian Infectious Diseases Society and the Canadian Thoracic Society. The Canadian Community-Acquired Pneumonia Working Group. Clin Infect Dis. 2000 Aug;31(2):383-421. Epub 2000 Sep 07. No abstract available. PMID: 10987698 [PubMed - indexed for MEDLINE] | |  | **4:** | [[No authors listed]](http://www.ncbi.nlm.nih.gov/entrez/query.fcgi?cmd=Retrieve&db=pubmed&dopt=Abstract&list_uids=10689421) | [Related Articles,](http://www.ncbi.nlm.nih.gov/entrez/query.fcgi?db=pubmed&cmd=Display&dopt=pubmed_pubmed&from_uid=10689421) 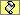[Links](javascript:PopUpMenu2_Set(Menu10689421);) | | --- | --- | --- | | [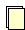](http://www.ncbi.nlm.nih.gov/entrez/query.fcgi?cmd=Retrieve&db=pubmed&dopt=Abstract&list_uids=10689421&itool=iconnoabstr) | Community-acquired pneumonia. Outpatient treatment of patients 16 years and older. Institute for Clinical Systems Improvement. Postgrad Med. 2000 Feb;107(2):246-53. No abstract available. PMID: 10689421 [PubMed - indexed for MEDLINE] | |  | **5:** | [[No authors listed]](http://www.ncbi.nlm.nih.gov/entrez/query.fcgi?cmd=Retrieve&db=pubmed&dopt=Abstract&list_uids=10199035) | [Related Articles,](http://www.ncbi.nlm.nih.gov/entrez/query.fcgi?db=pubmed&cmd=Display&dopt=pubmed_pubmed&from_uid=10199035) 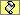[Links](javascript:PopUpMenu2_Set(Menu10199035);) | | --- | --- | --- | | [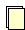](http://www.ncbi.nlm.nih.gov/entrez/query.fcgi?cmd=Retrieve&db=pubmed&dopt=Abstract&list_uids=10199035&itool=iconnoabstr) | [Treatment protocol for pneumonia in childhood. Work group "Pneumonias"] An Esp Pediatr. 1999 Feb;50(2):189-95. Spanish. No abstract available. PMID: 10199035 [PubMed - indexed for MEDLINE] | | |

- Spironolactone and heart failure:

| | [Entrez](http://www.ncbi.nlm.nih.gov/gquery/gquery.fcgi?itool=toolbar) | [PubMed](http://www.ncbi.nlm.nih.gov/entrez/query.fcgi?db=PubMed&itool=toolbar) | [Nucleotide](http://www.ncbi.nlm.nih.gov/entrez/query.fcgi?db=Nucleotide&itool=toolbar) | [Protein](http://www.ncbi.nlm.nih.gov/entrez/query.fcgi?db=Protein&itool=toolbar) | [Genome](http://www.ncbi.nlm.nih.gov/entrez/query.fcgi?db=Genome&itool=toolbar) | [Structure](http://www.ncbi.nlm.nih.gov/entrez/query.fcgi?db=Structure&itool=toolbar) | [OMIM](http://www.ncbi.nlm.nih.gov/entrez/query.fcgi?db=OMIM&itool=toolbar) | [PMC](http://www.ncbi.nlm.nih.gov/entrez/query.fcgi?db=PMC&itool=toolbar) | [Journals](http://www.ncbi.nlm.nih.gov/entrez/query.fcgi?db=Journals&itool=toolbar) | [Books](http://www.ncbi.nlm.nih.gov/entrez/query.fcgi?db=Books&itool=toolbar) | | --- | --- | --- | --- | --- | --- | --- | --- | --- | --- | |
| --- | --- | --- | --- | --- | --- | --- | --- | --- | --- | --- |
| | Search for | | --- | |
| |  | |  | [Limits](javascript:Go('Limits')) | [Preview/Index](javascript:Go('Index')) | [History](javascript:Go('History')) | [Clipboard](javascript:Go('Clipboard')) | [Details](javascript:Go('Details')) |  |  | | --- | --- | --- | --- | --- | --- | --- | --- | | | --- | --- | --- | --- | --- | --- | --- | --- | --- | --- | |

|  | | |
| --- | --- | --- |
| [About Entrez](http://www.ncbi.nlm.nih.gov/Database/index.html)  [Text Version](http://www.ncbi.nlm.nih.gov/entrez/queryd.fcgi?linkbar=plain)  Entrez PubMed  [Overview](http://www.ncbi.nlm.nih.gov/entrez/query/static/overview.html) [Help |](http://www.ncbi.nlm.nih.gov/entrez/query/static/help/pmhelp.html) [FAQ](http://www.ncbi.nlm.nih.gov/entrez/query/static/faq.html) [Tutorial](http://www.nlm.nih.gov/bsd/pubmed_tutorial/m1001.html) [New/Noteworthy](http://www.ncbi.nlm.nih.gov/entrez/query/static/new.html) [E-Utilities](http://eutils.ncbi.nlm.nih.gov/entrez/query/static/eutils_help.html)  PubMed Services [Journals Database](http://www.ncbi.nlm.nih.gov/entrez/query.fcgi?db=journals) [MeSH Database](http://www.ncbi.nlm.nih.gov/entrez/query.fcgi?db=mesh) [Single Citation Matcher](http://www.ncbi.nlm.nih.gov/entrez/query/static/citmatch.html) [Batch Citation Matcher](http://www.ncbi.nlm.nih.gov/entrez/getids.cgi) [Clinical Queries](http://www.ncbi.nlm.nih.gov/entrez/query/static/clinical.html) [LinkOut](http://www.ncbi.nlm.nih.gov/entrez/linkout) [Cubby](http://www.ncbi.nlm.nih.gov/entrez/cubby.fcgi?call=QueryExt.Query.last.Show&call=QueryExt.CubbyQuery..ShowAll)  Related Resources [Order Documents](http://www.nlm.nih.gov/loansomedoc/loansome_home.html) [NLM Catalog](http://www.ncbi.nlm.nih.gov/entrez/query.fcgi?db=nlmcatalog) [NLM Gateway](http://gateway.nlm.nih.gov/gw/Cmd) [TOXNET](http://toxnet.nlm.nih.gov/) [Consumer Health](http://www.nlm.nih.gov/medlineplus/) [Clinical Alerts](http://www.nlm.nih.gov/databases/alerts/clinical_alerts.html) [ClinicalTrials.gov](http://clinicaltrials.gov/ct/gui) [PubMed Central](http://www.pubmedcentral.nih.gov/) |  | | Field: **Title,** Limits: **Publication Date to 2002, Randomized Controlled Trial** | | --- |  | Show: | | --- | |  |  | Items 1 - 17 of 17 | One page. | | --- | --- |  | **1:** | [Rousseau MF, Gurne O, Duprez D, Van Mieghem W, Robert A, Ahn S, Galanti L, Ketelslegers JM; Belgian RALES Investigators.](http://www.ncbi.nlm.nih.gov/entrez/query.fcgi?cmd=Retrieve&db=pubmed&dopt=Abstract&list_uids=12427411) | [Related Articles,](http://www.ncbi.nlm.nih.gov/entrez/query.fcgi?db=pubmed&cmd=Display&dopt=pubmed_pubmed&from_uid=12427411) 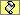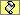[Links](javascript:PopUpMenu2_Set(Menu12427411);) | | --- | --- | --- | | [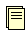](http://www.ncbi.nlm.nih.gov/entrez/query.fcgi?cmd=Retrieve&db=pubmed&dopt=Abstract&list_uids=12427411&itool=iconabstr) | Beneficial neurohormonal profile of spironolactone in severe congestive heart failure: results from the RALES neurohormonal substudy. J Am Coll Cardiol. 2002 Nov 6;40(9):1596-601.Erratum in: J Am Coll Cardiol. 2003 Nov 19;42(10):1865.  PMID: 12427411 [PubMed - indexed for MEDLINE] | |  | **2:** | [Kasama S, Toyama T, Kumakura H, Takayama Y, Ichikawa S, Suzuki T, Kurabayashi M.](http://www.ncbi.nlm.nih.gov/entrez/query.fcgi?cmd=Retrieve&db=pubmed&dopt=Abstract&list_uids=12368364) | [Related Articles,](http://www.ncbi.nlm.nih.gov/entrez/query.fcgi?db=pubmed&cmd=Display&dopt=pubmed_pubmed&from_uid=12368364) 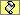[Links](javascript:PopUpMenu2_Set(Menu12368364);) | | --- | --- | --- | | [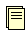](http://www.ncbi.nlm.nih.gov/entrez/query.fcgi?cmd=Retrieve&db=pubmed&dopt=Abstract&list_uids=12368364&itool=iconabstr) | Spironolactone improves cardiac sympathetic nerve activity and symptoms in patients with congestive heart failure. J Nucl Med. 2002 Oct;43(10):1279-85. PMID: 12368364 [PubMed - indexed for MEDLINE] | |  | **3:** | [Cicoira M, Zanolla L, Rossi A, Golia G, Franceschini L, Brighetti G, Marino P, Zardini P.](http://www.ncbi.nlm.nih.gov/entrez/query.fcgi?cmd=Retrieve&db=pubmed&dopt=Abstract&list_uids=12106936) | [Related Articles,](http://www.ncbi.nlm.nih.gov/entrez/query.fcgi?db=pubmed&cmd=Display&dopt=pubmed_pubmed&from_uid=12106936) 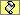[Links](javascript:PopUpMenu2_Set(Menu12106936);) | | --- | --- | --- | | [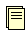](http://www.ncbi.nlm.nih.gov/entrez/query.fcgi?cmd=Retrieve&db=pubmed&dopt=Abstract&list_uids=12106936&itool=iconabstr) | Long-term, dose-dependent effects of spironolactone on left ventricular function and exercise tolerance in patients with chronic heart failure. J Am Coll Cardiol. 2002 Jul 17;40(2):304-10. PMID: 12106936 [PubMed - indexed for MEDLINE] | |  | **4:** | [Tsutamoto T, Wada A, Maeda K, Mabuchi N, Hayashi M, Tsutsui T, Ohnishi M, Sawaki M, Fujii M, Matsumoto T, Matsui T, Kinoshita M.](http://www.ncbi.nlm.nih.gov/entrez/query.fcgi?cmd=Retrieve&db=pubmed&dopt=Abstract&list_uids=11300427) | [Related Articles,](http://www.ncbi.nlm.nih.gov/entrez/query.fcgi?db=pubmed&cmd=Display&dopt=pubmed_pubmed&from_uid=11300427) 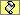[Links](javascript:PopUpMenu2_Set(Menu11300427);) | | --- | --- | --- | | [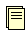](http://www.ncbi.nlm.nih.gov/entrez/query.fcgi?cmd=Retrieve&db=pubmed&dopt=Abstract&list_uids=11300427&itool=iconabstr) | Effect of spironolactone on plasma brain natriuretic peptide and left ventricular remodeling in patients with congestive heart failure. J Am Coll Cardiol. 2001 Apr;37(5):1228-33. PMID: 11300427 [PubMed - indexed for MEDLINE] | |  | **5:** | [Zannad F, Alla F, Dousset B, Perez A, Pitt B.](http://www.ncbi.nlm.nih.gov/entrez/query.fcgi?cmd=Retrieve&db=pubmed&dopt=Abstract&list_uids=11094035) | [Related Articles,](http://www.ncbi.nlm.nih.gov/entrez/query.fcgi?db=pubmed&cmd=Display&dopt=pubmed_pubmed&from_uid=11094035) 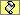[Links](javascript:PopUpMenu2_Set(Menu11094035);) | | --- | --- | --- | | [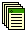](http://www.ncbi.nlm.nih.gov/entrez/query.fcgi?cmd=Retrieve&db=pubmed&dopt=Abstract&list_uids=11094035&itool=iconfft) | Limitation of excessive extracellular matrix turnover may contribute to survival benefit of spironolactone therapy in patients with congestive heart failure: insights from the randomized aldactone evaluation study (RALES). Rales Investigators. Circulation. 2000 Nov 28;102(22):2700-6.Erratum in: Circulation 2001 Jan 23;103(3):476.  PMID: 11094035 [PubMed - indexed for MEDLINE] | |  | **6:** | [Bednarz B, Cybulski J, Chamiec T.](http://www.ncbi.nlm.nih.gov/entrez/query.fcgi?cmd=Retrieve&db=pubmed&dopt=Abstract&list_uids=11081314) | [Related Articles,](http://www.ncbi.nlm.nih.gov/entrez/query.fcgi?db=pubmed&cmd=Display&dopt=pubmed_pubmed&from_uid=11081314) 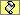[Links](javascript:PopUpMenu2_Set(Menu11081314);) | | --- | --- | --- | | [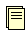](http://www.ncbi.nlm.nih.gov/entrez/query.fcgi?cmd=Retrieve&db=pubmed&dopt=Abstract&list_uids=11081314&itool=iconabstr) | [Comparison of the therapeutic efficacy of spironolactone and furosemide in patients with severe congestive heart failure] Pol Merkuriusz Lek. 2000 Aug;9(50):519-21. Polish. PMID: 11081314 [PubMed - indexed for MEDLINE] | |  | **7:** | [Ramires FJ, Mansur A, Coelho O, Maranhao M, Gruppi CJ, Mady C, Ramires JA.](http://www.ncbi.nlm.nih.gov/entrez/query.fcgi?cmd=Retrieve&db=pubmed&dopt=Abstract&list_uids=10802002) | [Related Articles,](http://www.ncbi.nlm.nih.gov/entrez/query.fcgi?db=pubmed&cmd=Display&dopt=pubmed_pubmed&from_uid=10802002) 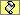[Links](javascript:PopUpMenu2_Set(Menu10802002);) | | --- | --- | --- | | [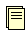](http://www.ncbi.nlm.nih.gov/entrez/query.fcgi?cmd=Retrieve&db=pubmed&dopt=Abstract&list_uids=10802002&itool=iconabstr) | Effect of spironolactone on ventricular arrhythmias in congestive heart failure secondary to idiopathic dilated or to ischemic cardiomyopathy. Am J Cardiol. 2000 May 15;85(10):1207-11. PMID: 10802002 [PubMed - indexed for MEDLINE] | |  | **8:** | [Georges B, Beguin C, Jadoul M.](http://www.ncbi.nlm.nih.gov/entrez/query.fcgi?cmd=Retrieve&db=pubmed&dopt=Abstract&list_uids=10776778) | [Related Articles,](http://www.ncbi.nlm.nih.gov/entrez/query.fcgi?db=pubmed&cmd=Display&dopt=pubmed_pubmed&from_uid=10776778) 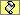[Links](javascript:PopUpMenu2_Set(Menu10776778);) | | --- | --- | --- | | [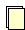](http://www.ncbi.nlm.nih.gov/entrez/query.fcgi?cmd=Retrieve&db=pubmed&dopt=Abstract&list_uids=10776778&itool=iconnoabstr) | Spironolactone and congestive heart-failure. Lancet. 2000 Apr 15;355(9212):1369-70. No abstract available. PMID: 10776778 [PubMed - indexed for MEDLINE] | |  | **9:** | [Farquharson CA, Struthers AD.](http://www.ncbi.nlm.nih.gov/entrez/query.fcgi?cmd=Retrieve&db=pubmed&dopt=Abstract&list_uids=10673249) | [Related Articles,](http://www.ncbi.nlm.nih.gov/entrez/query.fcgi?db=pubmed&cmd=Display&dopt=pubmed_pubmed&from_uid=10673249) 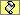[Links](javascript:PopUpMenu2_Set(Menu10673249);) | | --- | --- | --- | | [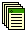](http://www.ncbi.nlm.nih.gov/entrez/query.fcgi?cmd=Retrieve&db=pubmed&dopt=Abstract&list_uids=10673249&itool=iconfft) | Spironolactone increases nitric oxide bioactivity, improves endothelial vasodilator dysfunction, and suppresses vascular angiotensin I/angiotensin II conversion in patients with chronic heart failure. Circulation. 2000 Feb 15;101(6):594-7. PMID: 10673249 [PubMed - indexed for MEDLINE] | |  | **10:** | [Lock M, Chun R.](http://www.ncbi.nlm.nih.gov/entrez/query.fcgi?cmd=Retrieve&db=pubmed&dopt=Abstract&list_uids=10587769) | [Related Articles,](http://www.ncbi.nlm.nih.gov/entrez/query.fcgi?db=pubmed&cmd=Display&dopt=pubmed_pubmed&from_uid=10587769) 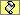[Links](javascript:PopUpMenu2_Set(Menu10587769);) | | --- | --- | --- | | [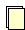](http://www.ncbi.nlm.nih.gov/entrez/query.fcgi?cmd=Retrieve&db=pubmed&dopt=Abstract&list_uids=10587769&itool=iconnoabstr) | What's old is new again. Spironolactone and heart failure. Can Fam Physician. 1999 Nov;45:2621-2. No abstract available. PMID: 10587769 [PubMed - indexed for MEDLINE] | |  | **11:** | [**Pitt B, Zannad F, Remme WJ, Cody R, Castaigne A, Perez A, Palensky J, Wittes J.**](http://www.ncbi.nlm.nih.gov/entrez/query.fcgi?cmd=Retrieve&db=pubmed&dopt=Abstract&list_uids=10471456) | [Related Articles,](http://www.ncbi.nlm.nih.gov/entrez/query.fcgi?db=pubmed&cmd=Display&dopt=pubmed_pubmed&from_uid=10471456) 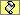[Links](javascript:PopUpMenu2_Set(Menu10471456);) | | --- | --- | --- | | [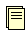](http://www.ncbi.nlm.nih.gov/entrez/query.fcgi?cmd=Retrieve&db=pubmed&dopt=Abstract&list_uids=10471456&itool=iconabstr) | **The effect of spironolactone on morbidity and mortality in patients with severe heart failure. Randomized Aldactone Evaluation Study Investigators.** N Engl J Med. 1999 Sep 2;341(10):709-17. PMID: 10471456 [PubMed - indexed for MEDLINE] | | |

- Converting enzyme inhibitors and heart failure:

| | [Entrez](http://www.ncbi.nlm.nih.gov/gquery/gquery.fcgi?itool=toolbar) | [PubMed](http://www.ncbi.nlm.nih.gov/entrez/query.fcgi?db=PubMed&itool=toolbar) | [Nucleotide](http://www.ncbi.nlm.nih.gov/entrez/query.fcgi?db=Nucleotide&itool=toolbar) | [Protein](http://www.ncbi.nlm.nih.gov/entrez/query.fcgi?db=Protein&itool=toolbar) | [Genome](http://www.ncbi.nlm.nih.gov/entrez/query.fcgi?db=Genome&itool=toolbar) | [Structure](http://www.ncbi.nlm.nih.gov/entrez/query.fcgi?db=Structure&itool=toolbar) | [OMIM](http://www.ncbi.nlm.nih.gov/entrez/query.fcgi?db=OMIM&itool=toolbar) | [PMC](http://www.ncbi.nlm.nih.gov/entrez/query.fcgi?db=PMC&itool=toolbar) | [Journals](http://www.ncbi.nlm.nih.gov/entrez/query.fcgi?db=Journals&itool=toolbar) | [Books](http://www.ncbi.nlm.nih.gov/entrez/query.fcgi?db=Books&itool=toolbar) | | --- | --- | --- | --- | --- | --- | --- | --- | --- | --- | |
| --- | --- | --- | --- | --- | --- | --- | --- | --- | --- | --- |
| | Search for | | --- | |
| |  | |  | [Limits](javascript:Go('Limits')) | [Preview/Index](javascript:Go('Index')) | [History](javascript:Go('History')) | [Clipboard](javascript:Go('Clipboard')) | [Details](javascript:Go('Details')) |  |  | | --- | --- | --- | --- | --- | --- | --- | --- | | | --- | --- | --- | --- | --- | --- | --- | --- | --- | --- | |

|  | | |
| --- | --- | --- |
| [About Entrez](http://www.ncbi.nlm.nih.gov/Database/index.html)  [Text Version](http://www.ncbi.nlm.nih.gov/entrez/queryd.fcgi?linkbar=plain)  Entrez PubMed  [Overview](http://www.ncbi.nlm.nih.gov/entrez/query/static/overview.html) [Help |](http://www.ncbi.nlm.nih.gov/entrez/query/static/help/pmhelp.html) [FAQ](http://www.ncbi.nlm.nih.gov/entrez/query/static/faq.html) [Tutorial](http://www.nlm.nih.gov/bsd/pubmed_tutorial/m1001.html) [New/Noteworthy](http://www.ncbi.nlm.nih.gov/entrez/query/static/new.html) [E-Utilities](http://eutils.ncbi.nlm.nih.gov/entrez/query/static/eutils_help.html)  PubMed Services [Journals Database](http://www.ncbi.nlm.nih.gov/entrez/query.fcgi?db=journals) [MeSH Database](http://www.ncbi.nlm.nih.gov/entrez/query.fcgi?db=mesh) [Single Citation Matcher](http://www.ncbi.nlm.nih.gov/entrez/query/static/citmatch.html) [Batch Citation Matcher](http://www.ncbi.nlm.nih.gov/entrez/getids.cgi) [Clinical Queries](http://www.ncbi.nlm.nih.gov/entrez/query/static/clinical.html) [LinkOut](http://www.ncbi.nlm.nih.gov/entrez/linkout) [Cubby](http://www.ncbi.nlm.nih.gov/entrez/cubby.fcgi?call=QueryExt.Query.last.Show&call=QueryExt.CubbyQuery..ShowAll)  Related Resources [Order Documents](http://www.nlm.nih.gov/loansomedoc/loansome_home.html) [NLM Catalog](http://www.ncbi.nlm.nih.gov/entrez/query.fcgi?db=nlmcatalog) [NLM Gateway](http://gateway.nlm.nih.gov/gw/Cmd) [TOXNET](http://toxnet.nlm.nih.gov/) [Consumer Health](http://www.nlm.nih.gov/medlineplus/) [Clinical Alerts](http://www.nlm.nih.gov/databases/alerts/clinical_alerts.html) [ClinicalTrials.gov](http://clinicaltrials.gov/ct/gui) [PubMed Central](http://www.pubmedcentral.nih.gov/) |  | | Field: **Title,** Limits: **All Child: 0-18 years, Publication Date to 2002** | | --- |  | Show: | | --- | |  |  | Items 1 - 4 of 4 | One page. | | --- | --- |  | **1:** | [Ajayi AA, Sofowora G, Balogun MO.](http://www.ncbi.nlm.nih.gov/entrez/query.fcgi?cmd=Retrieve&db=pubmed&dopt=Abstract&list_uids=9110062) | [Related Articles,](http://www.ncbi.nlm.nih.gov/entrez/query.fcgi?db=pubmed&cmd=Display&dopt=pubmed_pubmed&from_uid=9110062) 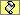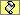[Links](javascript:PopUpMenu2_Set(Menu9110062);) | | --- | --- | --- | | [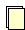](http://www.ncbi.nlm.nih.gov/entrez/query.fcgi?cmd=Retrieve&db=pubmed&dopt=Abstract&list_uids=9110062&itool=iconnoabstr) | Clinical implications of alpha 1 adrenergic blockade and angiotensin converting enzyme inhibition 'cross talk' in heart failure in Nigerians. Afr J Med Med Sci. 1996 Mar;25(1):95. No abstract available. PMID: 9110062 [PubMed - indexed for MEDLINE] | |  | **2:** | [Dutertre JP, Billaud EM, Autret E, Chantepie A, Oliver I, Laugier J.](http://www.ncbi.nlm.nih.gov/entrez/query.fcgi?cmd=Retrieve&db=pubmed&dopt=Abstract&list_uids=8512763) | [Related Articles,](http://www.ncbi.nlm.nih.gov/entrez/query.fcgi?db=pubmed&cmd=Display&dopt=pubmed_pubmed&from_uid=8512763) 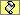[Links](javascript:PopUpMenu2_Set(Menu8512763);) | | --- | --- | --- | | [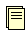](http://www.ncbi.nlm.nih.gov/entrez/query.fcgi?cmd=Retrieve&db=pubmed&dopt=Abstract&list_uids=8512763&itool=iconabstr) | Inhibition of angiotensin converting enzyme with enalapril maleate in infants with congestive heart failure. Br J Clin Pharmacol. 1993 May;35(5):528-30. PMID: 8512763 [PubMed - indexed for MEDLINE] | |  | **3:** | [Kleber FX, Niemoller L, Doering W.](http://www.ncbi.nlm.nih.gov/entrez/query.fcgi?cmd=Retrieve&db=pubmed&dopt=Abstract&list_uids=1389702) | [Related Articles,](http://www.ncbi.nlm.nih.gov/entrez/query.fcgi?db=pubmed&cmd=Display&dopt=pubmed_pubmed&from_uid=1389702) 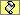[Links](javascript:PopUpMenu2_Set(Menu1389702);) | | --- | --- | --- | | [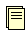](http://www.ncbi.nlm.nih.gov/entrez/query.fcgi?cmd=Retrieve&db=pubmed&dopt=Abstract&list_uids=1389702&itool=iconabstr) | Impact of converting enzyme inhibition on progression of chronic heart failure: results of the Munich Mild Heart Failure Trial. Br Heart J. 1992 Apr;67(4):289-96. PMID: 1389702 [PubMed - indexed for MEDLINE] | |  | **4:** | [**Rheuban KS, Carpenter MA, Ayers CA, Gutgesell HP.**](http://www.ncbi.nlm.nih.gov/entrez/query.fcgi?cmd=Retrieve&db=pubmed&dopt=Abstract&list_uids=2170613) | [Related Articles,](http://www.ncbi.nlm.nih.gov/entrez/query.fcgi?db=pubmed&cmd=Display&dopt=pubmed_pubmed&from_uid=2170613) 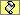[Links](javascript:PopUpMenu2_Set(Menu2170613);) | | --- | --- | --- | | [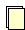](http://www.ncbi.nlm.nih.gov/entrez/query.fcgi?cmd=Retrieve&db=pubmed&dopt=Abstract&list_uids=2170613&itool=iconnoabstr) | **Acute hemodynamic effects of converting enzyme inhibition in infants with congestive heart failure.** J Pediatr. 1990 Oct;117(4):668-70. No abstract available. PMID: 2170613 [PubMed - indexed for MEDLINE] | | |

- Nosocomial pneumonia:

| | [Entrez](http://www.ncbi.nlm.nih.gov/gquery/gquery.fcgi?itool=toolbar) | [PubMed](http://www.ncbi.nlm.nih.gov/entrez/query.fcgi?db=PubMed&itool=toolbar) | [Nucleotide](http://www.ncbi.nlm.nih.gov/entrez/query.fcgi?db=Nucleotide&itool=toolbar) | [Protein](http://www.ncbi.nlm.nih.gov/entrez/query.fcgi?db=Protein&itool=toolbar) | [Genome](http://www.ncbi.nlm.nih.gov/entrez/query.fcgi?db=Genome&itool=toolbar) | [Structure](http://www.ncbi.nlm.nih.gov/entrez/query.fcgi?db=Structure&itool=toolbar) | [OMIM](http://www.ncbi.nlm.nih.gov/entrez/query.fcgi?db=OMIM&itool=toolbar) | [PMC](http://www.ncbi.nlm.nih.gov/entrez/query.fcgi?db=PMC&itool=toolbar) | [Journals](http://www.ncbi.nlm.nih.gov/entrez/query.fcgi?db=Journals&itool=toolbar) | [Books](http://www.ncbi.nlm.nih.gov/entrez/query.fcgi?db=Books&itool=toolbar) | | --- | --- | --- | --- | --- | --- | --- | --- | --- | --- | |
| --- | --- | --- | --- | --- | --- | --- | --- | --- | --- | --- |
| | Search for | | --- | |
| |  | |  | [Limits](javascript:Go('Limits')) | [Preview/Index](javascript:Go('Index')) | [History](javascript:Go('History')) | [Clipboard](javascript:Go('Clipboard')) | [Details](javascript:Go('Details')) |  |  | | --- | --- | --- | --- | --- | --- | --- | --- | | | --- | --- | --- | --- | --- | --- | --- | --- | --- | --- | |

|  | | |
| --- | --- | --- |
| [About Entrez](http://www.ncbi.nlm.nih.gov/Database/index.html)  [Text Version](http://www.ncbi.nlm.nih.gov/entrez/queryd.fcgi?linkbar=plain)  Entrez PubMed  [Overview](http://www.ncbi.nlm.nih.gov/entrez/query/static/overview.html) [Help |](http://www.ncbi.nlm.nih.gov/entrez/query/static/help/pmhelp.html) [FAQ](http://www.ncbi.nlm.nih.gov/entrez/query/static/faq.html) [Tutorial](http://www.nlm.nih.gov/bsd/pubmed_tutorial/m1001.html) [New/Noteworthy](http://www.ncbi.nlm.nih.gov/entrez/query/static/new.html) [E-Utilities](http://eutils.ncbi.nlm.nih.gov/entrez/query/static/eutils_help.html)  PubMed Services [Journals Database](http://www.ncbi.nlm.nih.gov/entrez/query.fcgi?db=journals) [MeSH Database](http://www.ncbi.nlm.nih.gov/entrez/query.fcgi?db=mesh) [Single Citation Matcher](http://www.ncbi.nlm.nih.gov/entrez/query/static/citmatch.html) [Batch Citation Matcher](http://www.ncbi.nlm.nih.gov/entrez/getids.cgi) [Clinical Queries](http://www.ncbi.nlm.nih.gov/entrez/query/static/clinical.html) [LinkOut](http://www.ncbi.nlm.nih.gov/entrez/linkout) [Cubby](http://www.ncbi.nlm.nih.gov/entrez/cubby.fcgi?call=QueryExt.Query.last.Show&call=QueryExt.CubbyQuery..ShowAll)  Related Resources [Order Documents](http://www.nlm.nih.gov/loansomedoc/loansome_home.html) [NLM Catalog](http://www.ncbi.nlm.nih.gov/entrez/query.fcgi?db=nlmcatalog) [NLM Gateway](http://gateway.nlm.nih.gov/gw/Cmd) [TOXNET](http://toxnet.nlm.nih.gov/) [Consumer Health](http://www.nlm.nih.gov/medlineplus/) [Clinical Alerts](http://www.nlm.nih.gov/databases/alerts/clinical_alerts.html) [ClinicalTrials.gov](http://clinicaltrials.gov/ct/gui) [PubMed Central](http://www.pubmedcentral.nih.gov/) |  | | Field: **Title,** Limits: **All Child: 0-18 years, Publication Date to 2002** | | --- |  | Show: | | --- | |  |  | Items 1 - 20 of 46 | |  | of 3 |  | [Next](javascript:var frm = document.frmQueryBox;frm.inputpage.value=2;Go('Pager');) | | --- | --- | --- | --- | | | --- | --- | --- | --- | --- | --- |  | **1:** | [Gil'manov AA, Vizel' AA, Malysheva IIu, Sorokina MSh, Sadykova RS, Buniatian AA.](http://www.ncbi.nlm.nih.gov/entrez/query.fcgi?cmd=Retrieve&db=pubmed&dopt=Abstract&list_uids=12561638) | [Related Articles,](http://www.ncbi.nlm.nih.gov/entrez/query.fcgi?db=pubmed&cmd=Display&dopt=pubmed_pubmed&from_uid=12561638) [Links](javascript:PopUpMenu2_Set(Menu12561638);) | | --- | --- | --- | |  | [Analysis of the efficiency of treatment and causes of death in nosocomial pneumonia in the Republic of Tatarstan] Probl Tuberk. 2002(7):26-30. Russian. PMID: 12561638 [PubMed - indexed for MEDLINE] | |  | **2:** | [Grudinina SA, Zubkov MM, Krotova LA, Kurdiukova IuP, Kutsenko MA, Marinin VF, Sidorenko SV, Sinpal'nikov AI, Solomatin AS, Sterkhova GV, Fesenko OV, Fomina IG, Chuchalin AG.](http://www.ncbi.nlm.nih.gov/entrez/query.fcgi?cmd=Retrieve&db=pubmed&dopt=Abstract&list_uids=12077934) | [Related Articles,](http://www.ncbi.nlm.nih.gov/entrez/query.fcgi?db=pubmed&cmd=Display&dopt=pubmed_pubmed&from_uid=12077934) [Links](javascript:PopUpMenu2_Set(Menu12077934);) | | --- | --- | --- | |  | [Comparison of linezolid and vancomycin in nosocomial pneumonia: results of the multicenter double-blind study] Antibiot Khimioter. 2002;47(1):12-7. Russian. PMID: 12077934 [PubMed - indexed for MEDLINE] | |  | **3:** | [Trubel HK, Meyer HG, Jahn B, Knuf M, Kamin W, Huth RG.](http://www.ncbi.nlm.nih.gov/entrez/query.fcgi?cmd=Retrieve&db=pubmed&dopt=Abstract&list_uids=12030402) | [Related Articles,](http://www.ncbi.nlm.nih.gov/entrez/query.fcgi?db=pubmed&cmd=Display&dopt=pubmed_pubmed&from_uid=12030402) [Links](javascript:PopUpMenu2_Set(Menu12030402);) | | --- | --- | --- | |  | Complicated nosocomial pneumonia due to Legionella pneumophila in an immunocompromised child. Scand J Infect Dis. 2002;34(3):219-21. PMID: 12030402 [PubMed - indexed for MEDLINE] | |  | **4:** | [Ewig S, Bauer T, Torres A.](http://www.ncbi.nlm.nih.gov/entrez/query.fcgi?cmd=Retrieve&db=pubmed&dopt=Abstract&list_uids=11923560) | [Related Articles,](http://www.ncbi.nlm.nih.gov/entrez/query.fcgi?db=pubmed&cmd=Display&dopt=pubmed_pubmed&from_uid=11923560) [Links](javascript:PopUpMenu2_Set(Menu11923560);) | | --- | --- | --- | |  | The pulmonary physician in critical care * 4: Nosocomial pneumonia. Thorax. 2002 Apr;57(4):366-71. Review. PMID: 11923560 [PubMed - indexed for MEDLINE] | |  | **5:** | [**Zar HJ, Cotton MF.**](http://www.ncbi.nlm.nih.gov/entrez/query.fcgi?cmd=Retrieve&db=pubmed&dopt=Abstract&list_uids=11888355) | [Related Articles,](http://www.ncbi.nlm.nih.gov/entrez/query.fcgi?db=pubmed&cmd=Display&dopt=pubmed_pubmed&from_uid=11888355) [Links](javascript:PopUpMenu2_Set(Menu11888355);) | | --- | --- | --- | |  | **Nosocomial pneumonia in pediatric patients: practical problems and rational solutions. Paediatr Drugs. 2002;4(2):73-83. Review.** PMID: 11888355 [PubMed - indexed for MEDLINE] | |  | **6:** | [Martinez-Aguilar G, Alpuche-Aranda CM, Anaya C, Alcantar-Curiel D, Gayosso C, Daza C, Mijares C, Tinoco JC, Santos JI.](http://www.ncbi.nlm.nih.gov/entrez/query.fcgi?cmd=Retrieve&db=pubmed&dopt=Abstract&list_uids=11842997) | [Related Articles,](http://www.ncbi.nlm.nih.gov/entrez/query.fcgi?db=pubmed&cmd=Display&dopt=pubmed_pubmed&from_uid=11842997) [Links](javascript:PopUpMenu2_Set(Menu11842997);) | | --- | --- | --- | |  | Outbreak of nosocomial sepsis and pneumonia in a newborn intensive care unit by multiresistant extended-spectrum beta-lactamase-producing Klebsiella pneumoniae: high impact on mortality. Infect Control Hosp Epidemiol. 2001 Nov;22(11):725-8. PMID: 11842997 [PubMed - indexed for MEDLINE] | | |

- Dilated cardiomyopathy and captopril:

| | :   | [Entrez](http://www.ncbi.nlm.nih.gov/gquery/gquery.fcgi?itool=toolbar) | [PubMed](http://www.ncbi.nlm.nih.gov/entrez/query.fcgi?db=PubMed&itool=toolbar) | [Nucleotide](http://www.ncbi.nlm.nih.gov/entrez/query.fcgi?db=Nucleotide&itool=toolbar) | [Protein](http://www.ncbi.nlm.nih.gov/entrez/query.fcgi?db=Protein&itool=toolbar) | [Genome](http://www.ncbi.nlm.nih.gov/entrez/query.fcgi?db=Genome&itool=toolbar) | [Structure](http://www.ncbi.nlm.nih.gov/entrez/query.fcgi?db=Structure&itool=toolbar) | [OMIM](http://www.ncbi.nlm.nih.gov/entrez/query.fcgi?db=OMIM&itool=toolbar) | [PMC](http://www.ncbi.nlm.nih.gov/entrez/query.fcgi?db=PMC&itool=toolbar) | [Journals](http://www.ncbi.nlm.nih.gov/entrez/query.fcgi?db=Journals&itool=toolbar) | [Books](http://www.ncbi.nlm.nih.gov/entrez/query.fcgi?db=Books&itool=toolbar) | | --- | --- | --- | --- | --- | --- | --- | --- | --- | --- | | | --- | --- | --- | --- | --- | --- | --- | --- | --- | --- | --- | | | Search for | | --- | | | |  | |  | [Limits](javascript:Go('Limits')) | [Preview/Index](javascript:Go('Index')) | [History](javascript:Go('History')) | [Clipboard](javascript:Go('Clipboard')) | [Details](javascript:Go('Details')) | | --- | --- | --- | --- | --- | --- | | | --- | --- | --- | --- | --- | --- | --- | --- | | | Field: **Title ,** Limits: **Publication Date to 2002** |  | Items 1 - 20 of 34 | |  | of 2 |  | [Next](javascript:var frm = document.frmQueryBox;frm.inputpage.value=2;Go('Pager');) | | --- | --- | --- | --- | | | --- | --- | --- | --- | --- | --- |  | **1:** | [Chen G, Lin LX, Zhuang WT, Yao J, Huang HB, Liang JX, Zhang FL, Wen JP, Li LT, Lin M, Lin QM.](http://www.ncbi.nlm.nih.gov/entrez/query.fcgi?cmd=Retrieve&db=pubmed&dopt=Abstract&list_uids=15257916) | [Related Articles,](http://www.ncbi.nlm.nih.gov/entrez/query.fcgi?db=pubmed&cmd=Display&dopt=pubmed_pubmed&from_uid=15257916) [Links](javascript:PopUpMenu2_Set(Menu15257916);) | | --- | --- | --- | |  | [Effects of captopril on myocardial tissue energy metabolism and inflammation in rats with diabetic cardiomyopathy] Di Yi Jun Yi Da Xue Xue Bao. 2004 Jul;24(7):827-8, 831. Chinese. PMID: 15257916 [PubMed - in process] | |  | **2:** | [Plante E, Gaudreau M, Lachance D, Drolet MC, Roussel E, Gauthier C, Lapointe E, Arsenault M, Couet J.](http://www.ncbi.nlm.nih.gov/entrez/query.fcgi?cmd=Retrieve&db=pubmed&dopt=Abstract&list_uids=15052285) | [Related Articles,](http://www.ncbi.nlm.nih.gov/entrez/query.fcgi?db=pubmed&cmd=Display&dopt=pubmed_pubmed&from_uid=15052285) [Links](javascript:PopUpMenu2_Set(Menu15052285);) | | --- | --- | --- | |  | Angiotensin-converting enzyme inhibitor captopril prevents volume overload cardiomyopathy in experimental chronic aortic valve regurgitation. Can J Physiol Pharmacol. 2004 Mar;82(3):191-9. PMID: 15052285 [PubMed - in process] | |  | **3:** | [Jansson K, Dahlstrom U, Karlberg KE, Karlsson E, Nyquist O, Nylander E.](http://www.ncbi.nlm.nih.gov/entrez/query.fcgi?cmd=Retrieve&db=pubmed&dopt=Abstract&list_uids=10935777) | [Related Articles,](http://www.ncbi.nlm.nih.gov/entrez/query.fcgi?db=pubmed&cmd=Display&dopt=pubmed_pubmed&from_uid=10935777) [Links](javascript:PopUpMenu2_Set(Menu10935777);) | | --- | --- | --- | |  | The value of repeated echocardiographic evaluation in patients with idiopathic dilated cardiomyopathy during treatment with metoprolol or captopril. Scand Cardiovasc J. 2000 Jun;34(3):293-300. PMID: 10935777 [PubMed - indexed for MEDLINE] | |  | **4:** | [Gvozdjakova A, Simko F, Kucharska J, Braunova Z, Psenek P, Kyselovic J.](http://www.ncbi.nlm.nih.gov/entrez/query.fcgi?cmd=Retrieve&db=pubmed&dopt=Abstract&list_uids=10475591) | [Related Articles,](http://www.ncbi.nlm.nih.gov/entrez/query.fcgi?db=pubmed&cmd=Display&dopt=pubmed_pubmed&from_uid=10475591) [Links](javascript:PopUpMenu2_Set(Menu10475591);) | | --- | --- | --- | |  | Captopril increased mitochondrial coenzyme Q10 level, improved respiratory chain function and energy production in the left ventricle in rabbits with smoke mitochondrial cardiomyopathy. Biofactors. 1999;10(1):61-5. PMID: 10475591 [PubMed - indexed for MEDLINE] | |  | **5:** | [Jansson K, Hagerman I, Ostlund R, Karlberg KE, Nylander E, Nyquist O, Dahlstrom U.](http://www.ncbi.nlm.nih.gov/entrez/query.fcgi?cmd=Retrieve&db=pubmed&dopt=Abstract&list_uids=10376178) | [Related Articles,](http://www.ncbi.nlm.nih.gov/entrez/query.fcgi?db=pubmed&cmd=Display&dopt=pubmed_pubmed&from_uid=10376178) [Links](javascript:PopUpMenu2_Set(Menu10376178);) | | --- | --- | --- | |  | The effects of metoprolol and captopril on heart rate variability in patients with idiopathic dilated cardiomyopathy. Clin Cardiol. 1999 Jun;22(6):397-402. PMID: 10376178 [PubMed - indexed for MEDLINE] | |  | **6:** | [Jansson K, Dahlstrom U, Karlberg BE, Karlsson E, Nylander E, Nyquist O, Karlberg KE.](http://www.ncbi.nlm.nih.gov/entrez/query.fcgi?cmd=Retrieve&db=pubmed&dopt=Abstract&list_uids=10363743) | [Related Articles,](http://www.ncbi.nlm.nih.gov/entrez/query.fcgi?db=pubmed&cmd=Display&dopt=pubmed_pubmed&from_uid=10363743) [Links](javascript:PopUpMenu2_Set(Menu10363743);) | | --- | --- | --- | |  | The circulating renin-angiotensin system during treatment with metoprolol or captopril in patients with heart failure due to non-ischaemic dilated cardiomyopathy. J Intern Med. 1999 May;245(5):435-43. PMID: 10363743 [PubMed - indexed for MEDLINE] | |  | **7:** | [Jansson K, Karlberg KE, Nylander E, Karlsson E, Nyquist O, Dahlstrom U.](http://www.ncbi.nlm.nih.gov/entrez/query.fcgi?cmd=Retrieve&db=pubmed&dopt=Abstract&list_uids=9243145) | [Related Articles,](http://www.ncbi.nlm.nih.gov/entrez/query.fcgi?db=pubmed&cmd=Display&dopt=pubmed_pubmed&from_uid=9243145) [Links](javascript:PopUpMenu2_Set(Menu9243145);) | | --- | --- | --- | |  | More favourable haemodynamic effects from metoprolol than from captopril in patients with dilated cardiomyopathy. Eur Heart J. 1997 Jul;18(7):1115-21. PMID: 9243145 [PubMed - indexed for MEDLINE] | |  | **8:** | [Shen W, Zhang X, Hu H, Gong L.](http://www.ncbi.nlm.nih.gov/entrez/query.fcgi?cmd=Retrieve&db=pubmed&dopt=Abstract&list_uids=8745586) | [Related Articles,](http://www.ncbi.nlm.nih.gov/entrez/query.fcgi?db=pubmed&cmd=Display&dopt=pubmed_pubmed&from_uid=8745586) [Links](javascript:PopUpMenu2_Set(Menu8745586);) | | --- | --- | --- | |  | Abnormal left ventricular systolic and diastolic functional response to isometric exercise in idiopathic dilated cardiomyopathy: beneficial effect of captopril. Chin Med Sci J. 1995 Dec;10(4):232-6. PMID: 8745586 [PubMed - indexed for MEDLINE] | |  | **9:** | [Kanda T, Araki M, Nakano M, Imai S, Suzuki T, Murata K, Kobayashi I.](http://www.ncbi.nlm.nih.gov/entrez/query.fcgi?cmd=Retrieve&db=pubmed&dopt=Abstract&list_uids=7752100) | [Related Articles,](http://www.ncbi.nlm.nih.gov/entrez/query.fcgi?db=pubmed&cmd=Display&dopt=pubmed_pubmed&from_uid=7752100) [Links](javascript:PopUpMenu2_Set(Menu7752100);) | | --- | --- | --- | |  | Chronic effect of losartan in a murine model of dilated cardiomyopathy: comparison with captopril. J Pharmacol Exp Ther. 1995 May;273(2):955-8. PMID: 7752100 [PubMed - indexed for MEDLINE] | |  | **10:** | [Jakob H, Sigmund M, Eschenhagen T, Mende U, Patten M, Schmitz W, Scholz H, Schulte am Esch J, Steinfath M, Hanrath P.](http://www.ncbi.nlm.nih.gov/entrez/query.fcgi?cmd=Retrieve&db=pubmed&dopt=Abstract&list_uids=7720758) | [Related Articles,](http://www.ncbi.nlm.nih.gov/entrez/query.fcgi?db=pubmed&cmd=Display&dopt=pubmed_pubmed&from_uid=7720758) [Links](javascript:PopUpMenu2_Set(Menu7720758);) | | --- | --- | --- | |  | Effect of captopril on myocardial beta-adrenoceptor density and Gi alpha-proteins in patients with mild to moderate heart failure due to dilated cardiomyopathy. Eur J Clin Pharmacol. 1995;47(5):389-94. PMID: 7720758 [PubMed - indexed for MEDLINE] | |  | **11:** | [Davison G, Hall CS, Miller JG, Scott M, Wickline SA.](http://www.ncbi.nlm.nih.gov/entrez/query.fcgi?cmd=Retrieve&db=pubmed&dopt=Abstract&list_uids=8087943) | [Related Articles,](http://www.ncbi.nlm.nih.gov/entrez/query.fcgi?db=pubmed&cmd=Display&dopt=pubmed_pubmed&from_uid=8087943) [Links](javascript:PopUpMenu2_Set(Menu8087943);) | | --- | --- | --- | |  | Cellular mechanisms of captopril-induced matrix remodeling in Syrian hamster cardiomyopathy. Circulation. 1994 Sep;90(3):1334-42. PMID: 8087943 [PubMed - indexed for MEDLINE] | |  | **12:** | [Keren G, Pardes A, Eschar Y, Koifman B, Scherez J, Geleranter I, Laniado S.](http://www.ncbi.nlm.nih.gov/entrez/query.fcgi?cmd=Retrieve&db=pubmed&dopt=Abstract&list_uids=8138400) | [Related Articles,](http://www.ncbi.nlm.nih.gov/entrez/query.fcgi?db=pubmed&cmd=Display&dopt=pubmed_pubmed&from_uid=8138400) [Links](javascript:PopUpMenu2_Set(Menu8138400);) | | --- | --- | --- | |  | One-year clinical and echocardiographic follow-up of patients with congestive cardiomyopathy treated with captopril compared to placebo. Isr J Med Sci. 1994 Jan;30(1):90-8. PMID: 8138400 [PubMed - indexed for MEDLINE] | |  | **13:** | [Salathe M, Weiss P, Ritz R.](http://www.ncbi.nlm.nih.gov/entrez/query.fcgi?cmd=Retrieve&db=pubmed&dopt=Abstract&list_uids=1467043) | [Related Articles,](http://www.ncbi.nlm.nih.gov/entrez/query.fcgi?db=pubmed&cmd=Display&dopt=pubmed_pubmed&from_uid=1467043) [Links](javascript:PopUpMenu2_Set(Menu1467043);) | | --- | --- | --- | |  | Rapid reversal of heart failure in a patient with phaeochromocytoma and catecholamine-induced cardiomyopathy who was treated with captopril. Br Heart J. 1992 Nov;68(5):527-8. PMID: 1467043 [PubMed - indexed for MEDLINE] | |  | **14:** | [Roberti RR, Martinez EE, Andrade JL, Araujo VL, Brito FS, Portugal OP, Horowitz SF.](http://www.ncbi.nlm.nih.gov/entrez/query.fcgi?cmd=Retrieve&db=pubmed&dopt=Abstract&list_uids=1644089) | [Related Articles,](http://www.ncbi.nlm.nih.gov/entrez/query.fcgi?db=pubmed&cmd=Display&dopt=pubmed_pubmed&from_uid=1644089) [Links](javascript:PopUpMenu2_Set(Menu1644089);) | | --- | --- | --- | |  | Chagas cardiomyopathy and captopril. Eur Heart J. 1992 Jul;13(7):966-70. PMID: 1644089 [PubMed - indexed for MEDLINE] | |  | **15:** | [Evangelista-Masip A, Bruguera-Cortada J, Serrat-Serradell R, Robles-Castro A, Galve-Basilio E, Alijarde-Guimera M, Soler-Soler J.](http://www.ncbi.nlm.nih.gov/entrez/query.fcgi?cmd=Retrieve&db=pubmed&dopt=Abstract&list_uids=1734651) | [Related Articles,](http://www.ncbi.nlm.nih.gov/entrez/query.fcgi?db=pubmed&cmd=Display&dopt=pubmed_pubmed&from_uid=1734651) [Links](javascript:PopUpMenu2_Set(Menu1734651);) | | --- | --- | --- | |  | Influence of mitral regurgitation on the response to captopril therapy for congestive heart failure caused by idiopathic dilated cardiomyopathy. Am J Cardiol. 1992 Feb 1;69(4):373-6. PMID: 1734651 [PubMed - indexed for MEDLINE] | |  | **16:** | [Keren G, Pardes A, Eschar Y, Hansch E, Scherez J, Laniado S.](http://www.ncbi.nlm.nih.gov/entrez/query.fcgi?cmd=Retrieve&db=pubmed&dopt=Abstract&list_uids=1301244) | [Related Articles,](http://www.ncbi.nlm.nih.gov/entrez/query.fcgi?db=pubmed&cmd=Display&dopt=pubmed_pubmed&from_uid=1301244) [Links](javascript:PopUpMenu2_Set(Menu1301244);) | | --- | --- | --- | |  | Left ventricular filling dynamics by Doppler echocardiography in dilated cardiomyopathy: one-year follow-up in patients treated with captopril compared to placebo. Cardiology. 1992;81(4-5):196-206. PMID: 1301244 [PubMed - indexed for MEDLINE] | |  | **17:** | [Latson LA.](http://www.ncbi.nlm.nih.gov/entrez/query.fcgi?cmd=Retrieve&db=pubmed&dopt=Abstract&list_uids=1991388) | [Related Articles,](http://www.ncbi.nlm.nih.gov/entrez/query.fcgi?db=pubmed&cmd=Display&dopt=pubmed_pubmed&from_uid=1991388) [Links](javascript:PopUpMenu2_Set(Menu1991388);) | | --- | --- | --- | |  | Captopril in children with cardiomyopathy. Circulation. 1991 Feb;83(2):707-8. No abstract available. PMID: 1991388 [PubMed - indexed for MEDLINE] | |  | **18:** | [**Bengur AR, Beekman RH, Rocchini AP, Crowley DC, Schork MA, Rosenthal A.**](http://www.ncbi.nlm.nih.gov/entrez/query.fcgi?cmd=Retrieve&db=pubmed&dopt=Abstract&list_uids=1991370) | [Related Articles,](http://www.ncbi.nlm.nih.gov/entrez/query.fcgi?db=pubmed&cmd=Display&dopt=pubmed_pubmed&from_uid=1991370) [Links](javascript:PopUpMenu2_Set(Menu1991370);) | | --- | --- | --- | |  | **Acute hemodynamic effects of captopril in children with a congestive or restrictive cardiomyopathy. Circulation. 1991 Feb;83(2):523-7.** PMID: 1991370 [PubMed - indexed for MEDLINE] | | |
| --- | --- | --- | --- | --- | --- | --- | --- | --- | --- | --- | --- | --- | --- | --- | --- | --- | --- | --- | --- | --- | --- | --- | --- | --- | --- | --- | --- | --- | --- | --- | --- | --- | --- | --- | --- | --- | --- | --- | --- | --- | --- | --- | --- | --- | --- | --- | --- | --- | --- | --- | --- | --- | --- | --- | --- | --- | --- | --- | --- | --- | --- | --- | --- | --- | --- | --- | --- | --- | --- | --- | --- | --- | --- | --- | --- | --- | --- | --- | --- | --- | --- | --- | --- | --- | --- | --- | --- | --- | --- | --- | --- | --- | --- | --- | --- | --- | --- | --- | --- | --- | --- | --- | --- | --- | --- | --- | --- | --- | --- | --- | --- | --- | --- | --- | --- | --- | --- | --- | --- | --- | --- | --- | --- | --- | --- | --- | --- | --- | --- | --- | --- | --- | --- | --- | --- | --- | --- |

- Bacterial meningitis:

| | [Entrez](http://www.ncbi.nlm.nih.gov/gquery/gquery.fcgi?itool=toolbar) | [PubMed](http://www.ncbi.nlm.nih.gov/entrez/query.fcgi?db=PubMed&itool=toolbar) | [Nucleotide](http://www.ncbi.nlm.nih.gov/entrez/query.fcgi?db=Nucleotide&itool=toolbar) | [Protein](http://www.ncbi.nlm.nih.gov/entrez/query.fcgi?db=Protein&itool=toolbar) | [Genome](http://www.ncbi.nlm.nih.gov/entrez/query.fcgi?db=Genome&itool=toolbar) | [Structure](http://www.ncbi.nlm.nih.gov/entrez/query.fcgi?db=Structure&itool=toolbar) | [OMIM](http://www.ncbi.nlm.nih.gov/entrez/query.fcgi?db=OMIM&itool=toolbar) | [PMC](http://www.ncbi.nlm.nih.gov/entrez/query.fcgi?db=PMC&itool=toolbar) | [Journals](http://www.ncbi.nlm.nih.gov/entrez/query.fcgi?db=Journals&itool=toolbar) | [Books](http://www.ncbi.nlm.nih.gov/entrez/query.fcgi?db=Books&itool=toolbar) | | --- | --- | --- | --- | --- | --- | --- | --- | --- | --- | |
| --- | --- | --- | --- | --- | --- | --- | --- | --- | --- | --- |
| | Search for | | --- | |
| |  | |  | [Limits](javascript:Go('Limits')) | [Preview/Index](javascript:Go('Index')) | [History](javascript:Go('History')) | [Clipboard](javascript:Go('Clipboard')) | [Details](javascript:Go('Details')) |  |  | | --- | --- | --- | --- | --- | --- | --- | --- | | | --- | --- | --- | --- | --- | --- | --- | --- | --- | --- | |

|  | | |
| --- | --- | --- |
| [About Entrez](http://www.ncbi.nlm.nih.gov/Database/index.html)  [Text Version](http://www.ncbi.nlm.nih.gov/entrez/queryd.fcgi?linkbar=plain)  Entrez PubMed  [Overview](http://www.ncbi.nlm.nih.gov/entrez/query/static/overview.html) [Help |](http://www.ncbi.nlm.nih.gov/entrez/query/static/help/pmhelp.html) [FAQ](http://www.ncbi.nlm.nih.gov/entrez/query/static/faq.html) [Tutorial](http://www.nlm.nih.gov/bsd/pubmed_tutorial/m1001.html) [New/Noteworthy](http://www.ncbi.nlm.nih.gov/entrez/query/static/new.html) [E-Utilities](http://eutils.ncbi.nlm.nih.gov/entrez/query/static/eutils_help.html)  PubMed Services [Journals Database](http://www.ncbi.nlm.nih.gov/entrez/query.fcgi?db=journals) [MeSH Database](http://www.ncbi.nlm.nih.gov/entrez/query.fcgi?db=mesh) [Single Citation Matcher](http://www.ncbi.nlm.nih.gov/entrez/query/static/citmatch.html) [Batch Citation Matcher](http://www.ncbi.nlm.nih.gov/entrez/getids.cgi) [Clinical Queries](http://www.ncbi.nlm.nih.gov/entrez/query/static/clinical.html) [LinkOut](http://www.ncbi.nlm.nih.gov/entrez/linkout) [Cubby](http://www.ncbi.nlm.nih.gov/entrez/cubby.fcgi?call=QueryExt.Query.last.Show&call=QueryExt.CubbyQuery..ShowAll)  Related Resources [Order Documents](http://www.nlm.nih.gov/loansomedoc/loansome_home.html) [NLM Catalog](http://www.ncbi.nlm.nih.gov/entrez/query.fcgi?db=nlmcatalog) [NLM Gateway](http://gateway.nlm.nih.gov/gw/Cmd) [TOXNET](http://toxnet.nlm.nih.gov/) [Consumer Health](http://www.nlm.nih.gov/medlineplus/) [Clinical Alerts](http://www.nlm.nih.gov/databases/alerts/clinical_alerts.html) [ClinicalTrials.gov](http://clinicaltrials.gov/ct/gui) [PubMed Central](http://www.pubmedcentral.nih.gov/) |  | | Field: **Title,** Limits: **All Child: 0-18 years, Publication Date to 2003, Review** | | --- |  | Show: | | --- | |  |  | Items 1 - 20 of 154 | |  | of 8 |  | [Next](javascript:var frm = document.frmQueryBox;frm.inputpage.value=2;Go('Pager');) | | --- | --- | --- | --- | | | --- | --- | --- | --- | --- | --- |  | **1:** | [Fuller DG, Duke T, Shann F, Curtis N.](http://www.ncbi.nlm.nih.gov/entrez/query.fcgi?cmd=Retrieve&db=pubmed&dopt=Abstract&list_uids=14738571) | [Related Articles,](http://www.ncbi.nlm.nih.gov/entrez/query.fcgi?db=pubmed&cmd=Display&dopt=pubmed_pubmed&from_uid=14738571) [Links](javascript:PopUpMenu2_Set(Menu14738571);) | | --- | --- | --- | |  | Antibiotic treatment for bacterial meningitis in children in developing countries. Ann Trop Paediatr. 2003 Dec;23(4):233-53. Review. PMID: 14738571 [PubMed - indexed for MEDLINE] | |  | **2:** | [Saitoh A, Beall B, Nizet V.](http://www.ncbi.nlm.nih.gov/entrez/query.fcgi?cmd=Retrieve&db=pubmed&dopt=Abstract&list_uids=14676492) | [Related Articles,](http://www.ncbi.nlm.nih.gov/entrez/query.fcgi?db=pubmed&cmd=Display&dopt=pubmed_pubmed&from_uid=14676492) [Links](javascript:PopUpMenu2_Set(Menu14676492);) | | --- | --- | --- | |  | Fulminant bacterial meningitis complicating sphenoid sinusitis. Pediatr Emerg Care. 2003 Dec;19(6):415-7. Review. No abstract available. PMID: 14676492 [PubMed - indexed for MEDLINE] | |  | **3:** | [van de Beek D, de Gans J, McIntyre P, Prasad K.](http://www.ncbi.nlm.nih.gov/entrez/query.fcgi?cmd=Retrieve&db=pubmed&dopt=Abstract&list_uids=12918010) | [Related Articles,](http://www.ncbi.nlm.nih.gov/entrez/query.fcgi?db=pubmed&cmd=Display&dopt=pubmed_pubmed&from_uid=12918010) [Links](javascript:PopUpMenu2_Set(Menu12918010);) | | --- | --- | --- | |  | Corticosteroids in acute bacterial meningitis. Cochrane Database Syst Rev. 2003(3):CD004305. Review. PMID: 12918010 [PubMed - indexed for MEDLINE] | |  | **4:** | [Duke T, Curtis N, Fuller DG.](http://www.ncbi.nlm.nih.gov/entrez/query.fcgi?cmd=Retrieve&db=pubmed&dopt=Abstract&list_uids=12877633) | [Related Articles,](http://www.ncbi.nlm.nih.gov/entrez/query.fcgi?db=pubmed&cmd=Display&dopt=pubmed_pubmed&from_uid=12877633) [Links](javascript:PopUpMenu2_Set(Menu12877633);) | | --- | --- | --- | |  | The management of bacterial meningitis in children. Expert Opin Pharmacother. 2003 Aug;4(8):1227-40. Review. PMID: 12877633 [PubMed - indexed for MEDLINE] | |  | **5:** | [Saez-Llorens X, McCracken GH Jr.](http://www.ncbi.nlm.nih.gov/entrez/query.fcgi?cmd=Retrieve&db=pubmed&dopt=Abstract&list_uids=12826449) | [Related Articles,](http://www.ncbi.nlm.nih.gov/entrez/query.fcgi?db=pubmed&cmd=Display&dopt=pubmed_pubmed&from_uid=12826449) [Links](javascript:PopUpMenu2_Set(Menu12826449);) | | --- | --- | --- | |  | Bacterial meningitis in children. Lancet. 2003 Jun 21;361(9375):2139-48. Review. PMID: 12826449 [PubMed - indexed for MEDLINE] | |  | **6:** | [**El Bashir H, Laundy M, Booy R.**](http://www.ncbi.nlm.nih.gov/entrez/query.fcgi?cmd=Retrieve&db=pubmed&dopt=Abstract&list_uids=12818910) | [Related Articles,](http://www.ncbi.nlm.nih.gov/entrez/query.fcgi?db=pubmed&cmd=Display&dopt=pubmed_pubmed&from_uid=12818910) [Links](javascript:PopUpMenu2_Set(Menu12818910);) | | --- | --- | --- | |  | **Diagnosis and treatment of bacterial meningitis. Arch Dis Child. 2003 Jul;88(7):615-20. Review.** PMID: 12818910 [PubMed - indexed for MEDLINE] | | |

- Pancreatitis:

| | [Entrez](http://www.ncbi.nlm.nih.gov/gquery/gquery.fcgi?itool=toolbar) | [PubMed](http://www.ncbi.nlm.nih.gov/entrez/query.fcgi?db=PubMed&itool=toolbar) | [Nucleotide](http://www.ncbi.nlm.nih.gov/entrez/query.fcgi?db=Nucleotide&itool=toolbar) | [Protein](http://www.ncbi.nlm.nih.gov/entrez/query.fcgi?db=Protein&itool=toolbar) | [Genome](http://www.ncbi.nlm.nih.gov/entrez/query.fcgi?db=Genome&itool=toolbar) | [Structure](http://www.ncbi.nlm.nih.gov/entrez/query.fcgi?db=Structure&itool=toolbar) | [OMIM](http://www.ncbi.nlm.nih.gov/entrez/query.fcgi?db=OMIM&itool=toolbar) | [PMC](http://www.ncbi.nlm.nih.gov/entrez/query.fcgi?db=PMC&itool=toolbar) | [Journals](http://www.ncbi.nlm.nih.gov/entrez/query.fcgi?db=Journals&itool=toolbar) | [Books](http://www.ncbi.nlm.nih.gov/entrez/query.fcgi?db=Books&itool=toolbar) | | --- | --- | --- | --- | --- | --- | --- | --- | --- | --- | |
| --- | --- | --- | --- | --- | --- | --- | --- | --- | --- | --- |
| | Search for | | --- | |
| |  | |  | [Limits](javascript:Go('Limits')) | [Preview/Index](javascript:Go('Index')) | [History](javascript:Go('History')) | [Clipboard](javascript:Go('Clipboard')) | [Details](javascript:Go('Details')) |  |  | | --- | --- | --- | --- | --- | --- | --- | --- | | | --- | --- | --- | --- | --- | --- | --- | --- | --- | --- | |

|  | | |
| --- | --- | --- |
| [About Entrez](http://www.ncbi.nlm.nih.gov/Database/index.html)  [Text Version](http://www.ncbi.nlm.nih.gov/entrez/queryd.fcgi?linkbar=plain)  Entrez PubMed  [Overview](http://www.ncbi.nlm.nih.gov/entrez/query/static/overview.html) [Help |](http://www.ncbi.nlm.nih.gov/entrez/query/static/help/pmhelp.html) [FAQ](http://www.ncbi.nlm.nih.gov/entrez/query/static/faq.html) [Tutorial](http://www.nlm.nih.gov/bsd/pubmed_tutorial/m1001.html) [New/Noteworthy](http://www.ncbi.nlm.nih.gov/entrez/query/static/new.html) [E-Utilities](http://eutils.ncbi.nlm.nih.gov/entrez/query/static/eutils_help.html)  PubMed Services [Journals Database](http://www.ncbi.nlm.nih.gov/entrez/query.fcgi?db=journals) [MeSH Database](http://www.ncbi.nlm.nih.gov/entrez/query.fcgi?db=mesh) [Single Citation Matcher](http://www.ncbi.nlm.nih.gov/entrez/query/static/citmatch.html) [Batch Citation Matcher](http://www.ncbi.nlm.nih.gov/entrez/getids.cgi) [Clinical Queries](http://www.ncbi.nlm.nih.gov/entrez/query/static/clinical.html) [LinkOut](http://www.ncbi.nlm.nih.gov/entrez/linkout) [Cubby](http://www.ncbi.nlm.nih.gov/entrez/cubby.fcgi?call=QueryExt.Query.last.Show&call=QueryExt.CubbyQuery..ShowAll)  Related Resources [Order Documents](http://www.nlm.nih.gov/loansomedoc/loansome_home.html) [NLM Catalog](http://www.ncbi.nlm.nih.gov/entrez/query.fcgi?db=nlmcatalog) [NLM Gateway](http://gateway.nlm.nih.gov/gw/Cmd) [TOXNET](http://toxnet.nlm.nih.gov/) [Consumer Health](http://www.nlm.nih.gov/medlineplus/) [Clinical Alerts](http://www.nlm.nih.gov/databases/alerts/clinical_alerts.html) [ClinicalTrials.gov](http://clinicaltrials.gov/ct/gui) [PubMed Central](http://www.pubmedcentral.nih.gov/) |  | | Field: **Title,** Limits: **All Child: 0-18 years, Publication Date to 2002, Review** | | --- |  | Show: | | --- | |  |  | Items 1 - 20 of 102 | |  | of 6 |  | [Next](javascript:var frm = document.frmQueryBox;frm.inputpage.value=2;Go('Pager');) | | --- | --- | --- | --- | | | --- | --- | --- | --- | --- | --- |  | **1:** | [Davenport M.](http://www.ncbi.nlm.nih.gov/entrez/query.fcgi?cmd=Retrieve&db=pubmed&dopt=Abstract&list_uids=12420914) | [Related Articles,](http://www.ncbi.nlm.nih.gov/entrez/query.fcgi?db=pubmed&cmd=Display&dopt=pubmed_pubmed&from_uid=12420914) [Links](javascript:PopUpMenu2_Set(Menu12420914);) | | --- | --- | --- | |  | Acute and chronic pancreatitis. Indian J Pediatr. 2002 Sep;69(9):801-7. Review. PMID: 12420914 [PubMed - indexed for MEDLINE] | |  | **2:** | [Saisho H, Yamaguchi T, Ishihara T.](http://www.ncbi.nlm.nih.gov/entrez/query.fcgi?cmd=Retrieve&db=pubmed&dopt=Abstract&list_uids=12415853) | [Related Articles,](http://www.ncbi.nlm.nih.gov/entrez/query.fcgi?db=pubmed&cmd=Display&dopt=pubmed_pubmed&from_uid=12415853) [Links](javascript:PopUpMenu2_Set(Menu12415853);) | | --- | --- | --- | |  | [Non-surgical treatment of pancreatic stones in patients with chronic pancreatitis and pain] Nippon Shokakibyo Gakkai Zasshi. 2002 Oct;99(10):1186-90. Review. Japanese. No abstract available. PMID: 12415853 [PubMed - indexed for MEDLINE] | |  | **3:** | [Cohn JA, Noone PG, Jowell PS.](http://www.ncbi.nlm.nih.gov/entrez/query.fcgi?cmd=Retrieve&db=pubmed&dopt=Abstract&list_uids=12227654) | [Related Articles,](http://www.ncbi.nlm.nih.gov/entrez/query.fcgi?db=pubmed&cmd=Display&dopt=pubmed_pubmed&from_uid=12227654) [Links](javascript:PopUpMenu2_Set(Menu12227654);) | | --- | --- | --- | |  | Idiopathic pancreatitis related to CFTR: complex inheritance and identification of a modifier gene. J Investig Med. 2002 Sep;50(5):247S-255S. Review. PMID: 12227654 [PubMed - indexed for MEDLINE] | |  | **4:** | [Chavez M, Nago A.](http://www.ncbi.nlm.nih.gov/entrez/query.fcgi?cmd=Retrieve&db=pubmed&dopt=Abstract&list_uids=12165785) | [Related Articles,](http://www.ncbi.nlm.nih.gov/entrez/query.fcgi?db=pubmed&cmd=Display&dopt=pubmed_pubmed&from_uid=12165785) [Links](javascript:PopUpMenu2_Set(Menu12165785);) | | --- | --- | --- | |  | [Acute pancreatitis: use of a new prognostic stage system] Rev Gastroenterol Peru. 1996 Sep-Dec;16(3):208-13. Review. Spanish. PMID: 12165785 [PubMed - indexed for MEDLINE] | |  | **5:** | [Witt H.](http://www.ncbi.nlm.nih.gov/entrez/query.fcgi?cmd=Retrieve&db=pubmed&dopt=Abstract&list_uids=12120220) | [Related Articles,](http://www.ncbi.nlm.nih.gov/entrez/query.fcgi?db=pubmed&cmd=Display&dopt=pubmed_pubmed&from_uid=12120220) [Links](javascript:PopUpMenu2_Set(Menu12120220);) | | --- | --- | --- | |  | Gene mutations in children with chronic pancreatitis. Pancreatology. 2001;1(5):432-8. Review. PMID: 12120220 [PubMed - indexed for MEDLINE] | |  | **6:** | [Ellis I, Lerch MM, Whitcomb DC; Consensus Committees of the European Registry of Hereditary Pancreatic Diseases, Midwest Multi-Center Pancreatic Study Group, International Association of Pancreatology.](http://www.ncbi.nlm.nih.gov/entrez/query.fcgi?cmd=Retrieve&db=pubmed&dopt=Abstract&list_uids=12120217) | [Related Articles,](http://www.ncbi.nlm.nih.gov/entrez/query.fcgi?db=pubmed&cmd=Display&dopt=pubmed_pubmed&from_uid=12120217) [Links](javascript:PopUpMenu2_Set(Menu12120217);) | | --- | --- | --- | |  | Genetic testing for hereditary pancreatitis: guidelines for indications, counselling, consent and privacy issues. Pancreatology. 2001;1(5):405-15. Review. No abstract available. PMID: 12120217 [PubMed - indexed for MEDLINE] | |  | **7:** | [Sclabas G, Kirschstein T, Uhl W, Hurlimann R, Ruchti C, Buchler MW.](http://www.ncbi.nlm.nih.gov/entrez/query.fcgi?cmd=Retrieve&db=pubmed&dopt=Abstract&list_uids=12064796) | [Related Articles,](http://www.ncbi.nlm.nih.gov/entrez/query.fcgi?db=pubmed&cmd=Display&dopt=pubmed_pubmed&from_uid=12064796) [Links](javascript:PopUpMenu2_Set(Menu12064796);) | | --- | --- | --- | |  | Juvenile idiopathic fibrosing pancreatitis. Dig Dis Sci. 2002 Jun;47(6):1230-5. Review. PMID: 12064796 [PubMed - indexed for MEDLINE] | |  | **8:** | [Witt H, Becker M.](http://www.ncbi.nlm.nih.gov/entrez/query.fcgi?cmd=Retrieve&db=pubmed&dopt=Abstract&list_uids=11840029) | [Related Articles,](http://www.ncbi.nlm.nih.gov/entrez/query.fcgi?db=pubmed&cmd=Display&dopt=pubmed_pubmed&from_uid=11840029) [Links](javascript:PopUpMenu2_Set(Menu11840029);) | | --- | --- | --- | |  | Genetics of chronic pancreatitis. J Pediatr Gastroenterol Nutr. 2002 Feb;34(2):125-36. Review. No abstract available. PMID: 11840029 [PubMed - indexed for MEDLINE] | |  | **9:** | [Jackson WD.](http://www.ncbi.nlm.nih.gov/entrez/query.fcgi?cmd=Retrieve&db=pubmed&dopt=Abstract&list_uids=11801891) | [Related Articles,](http://www.ncbi.nlm.nih.gov/entrez/query.fcgi?db=pubmed&cmd=Display&dopt=pubmed_pubmed&from_uid=11801891) [Links](javascript:PopUpMenu2_Set(Menu11801891);) | | --- | --- | --- | |  | Pancreatitis: etiology, diagnosis, and management. Curr Opin Pediatr. 2001 Oct;13(5):447-51. Review. PMID: 11801891 [PubMed - indexed for MEDLINE] | |  | **10:** | [Rosell Camps A, Hervas Palazon J, Ramos Asensio R, Henales Villate V, Alonso Sainz F.](http://www.ncbi.nlm.nih.gov/entrez/query.fcgi?cmd=Retrieve&db=pubmed&dopt=Abstract&list_uids=11488112) | [Related Articles,](http://www.ncbi.nlm.nih.gov/entrez/query.fcgi?db=pubmed&cmd=Display&dopt=pubmed_pubmed&from_uid=11488112) [Links](javascript:PopUpMenu2_Set(Menu11488112);) | | --- | --- | --- | |  | [Idiopathic fibrosing pancreatitis in pediatrics] Rev Esp Enferm Dig. 2001 May;93(5):332-4. Review. Spanish. No abstract available. PMID: 11488112 [PubMed - indexed for MEDLINE] | |  | **11:** | [Tarasenko VS, Nikitenko VI, Kubyshkln VA.](http://www.ncbi.nlm.nih.gov/entrez/query.fcgi?cmd=Retrieve&db=pubmed&dopt=Abstract&list_uids=11209244) | [Related Articles,](http://www.ncbi.nlm.nih.gov/entrez/query.fcgi?db=pubmed&cmd=Display&dopt=pubmed_pubmed&from_uid=11209244) [Links](javascript:PopUpMenu2_Set(Menu11209244);) | | --- | --- | --- | |  | [Acute pancreatitis and bacterial translocation] Vestn Khir Im I I Grek. 2000;159(6):86-9. Review. Russian. No abstract available. PMID: 11209244 [PubMed - indexed for MEDLINE] | |  | **12:** | [Drenth JP, Jansen JB.](http://www.ncbi.nlm.nih.gov/entrez/query.fcgi?cmd=Retrieve&db=pubmed&dopt=Abstract&list_uids=11143296) | [Related Articles,](http://www.ncbi.nlm.nih.gov/entrez/query.fcgi?db=pubmed&cmd=Display&dopt=pubmed_pubmed&from_uid=11143296) [Links](javascript:PopUpMenu2_Set(Menu11143296);) | | --- | --- | --- | |  | [From gene to disease; hereditary pancreatitis] Ned Tijdschr Geneeskd. 2000 Nov 25;144(48):2301-2. Review. Dutch. PMID: 11143296 [PubMed - indexed for MEDLINE] | |  | **13:** | [Mehta DI.](http://www.ncbi.nlm.nih.gov/entrez/query.fcgi?cmd=Retrieve&db=pubmed&dopt=Abstract&list_uids=11132475) | [Related Articles,](http://www.ncbi.nlm.nih.gov/entrez/query.fcgi?db=pubmed&cmd=Display&dopt=pubmed_pubmed&from_uid=11132475) [Links](javascript:PopUpMenu2_Set(Menu11132475);) | | --- | --- | --- | |  | Acute and chronic pancreatitis in childhood. Indian J Pediatr. 1999;66(1 Suppl):S81-6. Review. PMID: 11132475 [PubMed - indexed for MEDLINE] | |  | **14:** | [**Pietzak MM, Thomas DW.**](http://www.ncbi.nlm.nih.gov/entrez/query.fcgi?cmd=Retrieve&db=pubmed&dopt=Abstract&list_uids=11121497) | [Related Articles,](http://www.ncbi.nlm.nih.gov/entrez/query.fcgi?db=pubmed&cmd=Display&dopt=pubmed_pubmed&from_uid=11121497) [Links](javascript:PopUpMenu2_Set(Menu11121497);) | | --- | --- | --- | |  | **Pancreatitis in childhood. Pediatr Rev. 2000 Dec;21(12):406-12. Review. No abstract available.** PMID: 11121497 [PubMed - indexed for MEDLINE] | | |

- - Pancreatitis:

| | [Entrez](http://www.ncbi.nlm.nih.gov/gquery/gquery.fcgi?itool=toolbar) | [PubMed](http://www.ncbi.nlm.nih.gov/entrez/query.fcgi?db=PubMed&itool=toolbar) | [Nucleotide](http://www.ncbi.nlm.nih.gov/entrez/query.fcgi?db=Nucleotide&itool=toolbar) | [Protein](http://www.ncbi.nlm.nih.gov/entrez/query.fcgi?db=Protein&itool=toolbar) | [Genome](http://www.ncbi.nlm.nih.gov/entrez/query.fcgi?db=Genome&itool=toolbar) | [Structure](http://www.ncbi.nlm.nih.gov/entrez/query.fcgi?db=Structure&itool=toolbar) | [OMIM](http://www.ncbi.nlm.nih.gov/entrez/query.fcgi?db=OMIM&itool=toolbar) | [PMC](http://www.ncbi.nlm.nih.gov/entrez/query.fcgi?db=PMC&itool=toolbar) | [Journals](http://www.ncbi.nlm.nih.gov/entrez/query.fcgi?db=Journals&itool=toolbar) | [Books](http://www.ncbi.nlm.nih.gov/entrez/query.fcgi?db=Books&itool=toolbar) | | --- | --- | --- | --- | --- | --- | --- | --- | --- | --- | |
| --- | --- | --- | --- | --- | --- | --- | --- | --- | --- | --- |
| | Search for | | --- | |
| |  | |  | [Limits](javascript:Go('Limits')) | [Preview/Index](javascript:Go('Index')) | [History](javascript:Go('History')) | [Clipboard](javascript:Go('Clipboard')) | [Details](javascript:Go('Details')) |  |  | | --- | --- | --- | --- | --- | --- | --- | --- | | | --- | --- | --- | --- | --- | --- | --- | --- | --- | --- | |

|  | | |
| --- | --- | --- |
| [About Entrez](http://www.ncbi.nlm.nih.gov/Database/index.html)  [Text Version](http://www.ncbi.nlm.nih.gov/entrez/queryd.fcgi?linkbar=plain)  Entrez PubMed  [Overview](http://www.ncbi.nlm.nih.gov/entrez/query/static/overview.html) [Help |](http://www.ncbi.nlm.nih.gov/entrez/query/static/help/pmhelp.html) [FAQ](http://www.ncbi.nlm.nih.gov/entrez/query/static/faq.html) [Tutorial](http://www.nlm.nih.gov/bsd/pubmed_tutorial/m1001.html) [New/Noteworthy](http://www.ncbi.nlm.nih.gov/entrez/query/static/new.html) [E-Utilities](http://eutils.ncbi.nlm.nih.gov/entrez/query/static/eutils_help.html)  PubMed Services [Journals Database](http://www.ncbi.nlm.nih.gov/entrez/query.fcgi?db=journals) [MeSH Database](http://www.ncbi.nlm.nih.gov/entrez/query.fcgi?db=mesh) [Single Citation Matcher](http://www.ncbi.nlm.nih.gov/entrez/query/static/citmatch.html) [Batch Citation Matcher](http://www.ncbi.nlm.nih.gov/entrez/getids.cgi) [Clinical Queries](http://www.ncbi.nlm.nih.gov/entrez/query/static/clinical.html) [LinkOut](http://www.ncbi.nlm.nih.gov/entrez/linkout) [Cubby](http://www.ncbi.nlm.nih.gov/entrez/cubby.fcgi?call=QueryExt.Query.last.Show&call=QueryExt.CubbyQuery..ShowAll)  Related Resources [Order Documents](http://www.nlm.nih.gov/loansomedoc/loansome_home.html) [NLM Catalog](http://www.ncbi.nlm.nih.gov/entrez/query.fcgi?db=nlmcatalog) [NLM Gateway](http://gateway.nlm.nih.gov/gw/Cmd) [TOXNET](http://toxnet.nlm.nih.gov/) [Consumer Health](http://www.nlm.nih.gov/medlineplus/) [Clinical Alerts](http://www.nlm.nih.gov/databases/alerts/clinical_alerts.html) [ClinicalTrials.gov](http://clinicaltrials.gov/ct/gui) [PubMed Central](http://www.pubmedcentral.nih.gov/) |  | | Field: **Title,** Limits: **Publication Date to 2002, Practice Guideline** | | --- |  | Show: | | --- | |  |  | Items 1 - 12 of 12 | One page. | | --- | --- |  | **1:** | [**Mayumi T, Ura H, Arata S, Kitamura N, Kiriyama I, Shibuya K, Sekimoto M, Nago N, Hirota M, Yoshida M, Ito Y, Hirata K, Takada T; Working Group for the Practical Guidelines for Acute Pancreatitis. Japanese Society of Emergency Abdominal Medicine.**](http://www.ncbi.nlm.nih.gov/entrez/query.fcgi?cmd=Retrieve&db=pubmed&dopt=Abstract&list_uids=12483262) | [Related Articles,](http://www.ncbi.nlm.nih.gov/entrez/query.fcgi?db=pubmed&cmd=Display&dopt=pubmed_pubmed&from_uid=12483262) [Links](javascript:PopUpMenu2_Set(Menu12483262);) | | --- | --- | --- | |  | **Evidence-based clinical practice guidelines for acute pancreatitis: proposals. J Hepatobiliary Pancreat Surg. 2002;9(4):413-22.** PMID: 12483262 [PubMed - indexed for MEDLINE] | |  | **2:** | [Uhl W, Warshaw A, Imrie C, Bassi C, McKay CJ, Lankisch PG, Carter R, Di Magno E, Banks PA, Whitcomb DC, Dervenis C, Ulrich CD, Satake K, Ghaneh P, Hartwig W, Werner J, McEntee G, Neoptolemos JP, Buchler MW; International Association of Pancreatology.](http://www.ncbi.nlm.nih.gov/entrez/query.fcgi?cmd=Retrieve&db=pubmed&dopt=Abstract&list_uids=12435871) | [Related Articles,](http://www.ncbi.nlm.nih.gov/entrez/query.fcgi?db=pubmed&cmd=Display&dopt=pubmed_pubmed&from_uid=12435871) [Links](javascript:PopUpMenu2_Set(Menu12435871);) | | --- | --- | --- | |  | IAP Guidelines for the Surgical Management of Acute Pancreatitis. Pancreatology. 2002;2(6):565-73. PMID: 12435871 [PubMed - indexed for MEDLINE] | |  | **3:** | [Meier R, Beglinger C, Layer P, Gullo L, Keim V, Laugier R, Friess H, Schweitzer M, Macfie J; ESPEN Consensus Group.](http://www.ncbi.nlm.nih.gov/entrez/query.fcgi?cmd=Retrieve&db=pubmed&dopt=Abstract&list_uids=12056792) | [Related Articles,](http://www.ncbi.nlm.nih.gov/entrez/query.fcgi?db=pubmed&cmd=Display&dopt=pubmed_pubmed&from_uid=12056792) [Links](javascript:PopUpMenu2_Set(Menu12056792);) | | --- | --- | --- | |  | ESPEN guidelines on nutrition in acute pancreatitis. European Society of Parenteral and Enteral Nutrition. Clin Nutr. 2002 Apr;21(2):173-83. Review. No abstract available. PMID: 12056792 [PubMed - indexed for MEDLINE] | | |

- Hydrocarbon ingestion: :

| | [Entrez](http://www.ncbi.nlm.nih.gov/gquery/gquery.fcgi?itool=toolbar) | [PubMed](http://www.ncbi.nlm.nih.gov/entrez/query.fcgi?db=PubMed&itool=toolbar) | [Nucleotide](http://www.ncbi.nlm.nih.gov/entrez/query.fcgi?db=Nucleotide&itool=toolbar) | [Protein](http://www.ncbi.nlm.nih.gov/entrez/query.fcgi?db=Protein&itool=toolbar) | [Genome](http://www.ncbi.nlm.nih.gov/entrez/query.fcgi?db=Genome&itool=toolbar) | [Structure](http://www.ncbi.nlm.nih.gov/entrez/query.fcgi?db=Structure&itool=toolbar) | [OMIM](http://www.ncbi.nlm.nih.gov/entrez/query.fcgi?db=OMIM&itool=toolbar) | [PMC](http://www.ncbi.nlm.nih.gov/entrez/query.fcgi?db=PMC&itool=toolbar) | [Journals](http://www.ncbi.nlm.nih.gov/entrez/query.fcgi?db=Journals&itool=toolbar) | [Books](http://www.ncbi.nlm.nih.gov/entrez/query.fcgi?db=Books&itool=toolbar) | | --- | --- | --- | --- | --- | --- | --- | --- | --- | --- | |
| --- | --- | --- | --- | --- | --- | --- | --- | --- | --- | --- |
| | Search for | | --- | |
| |  | |  | [Limits](javascript:Go('Limits')) | [Preview/Index](javascript:Go('Index')) | [History](javascript:Go('History')) | [Clipboard](javascript:Go('Clipboard')) | [Details](javascript:Go('Details')) |  |  | | --- | --- | --- | --- | --- | --- | --- | --- | | | --- | --- | --- | --- | --- | --- | --- | --- | --- | --- | |

|  | | |
| --- | --- | --- |
| [About Entrez](http://www.ncbi.nlm.nih.gov/Database/index.html)  [Text Version](http://www.ncbi.nlm.nih.gov/entrez/queryd.fcgi?linkbar=plain)  Entrez PubMed  [Overview](http://www.ncbi.nlm.nih.gov/entrez/query/static/overview.html) [Help |](http://www.ncbi.nlm.nih.gov/entrez/query/static/help/pmhelp.html) [FAQ](http://www.ncbi.nlm.nih.gov/entrez/query/static/faq.html) [Tutorial](http://www.nlm.nih.gov/bsd/pubmed_tutorial/m1001.html) [New/Noteworthy](http://www.ncbi.nlm.nih.gov/entrez/query/static/new.html) [E-Utilities](http://eutils.ncbi.nlm.nih.gov/entrez/query/static/eutils_help.html)  PubMed Services [Journals Database](http://www.ncbi.nlm.nih.gov/entrez/query.fcgi?db=journals) [MeSH Database](http://www.ncbi.nlm.nih.gov/entrez/query.fcgi?db=mesh) [Single Citation Matcher](http://www.ncbi.nlm.nih.gov/entrez/query/static/citmatch.html) [Batch Citation Matcher](http://www.ncbi.nlm.nih.gov/entrez/getids.cgi) [Clinical Queries](http://www.ncbi.nlm.nih.gov/entrez/query/static/clinical.html) [LinkOut](http://www.ncbi.nlm.nih.gov/entrez/linkout) [Cubby](http://www.ncbi.nlm.nih.gov/entrez/cubby.fcgi?call=QueryExt.Query.last.Show&call=QueryExt.CubbyQuery..ShowAll)  Related Resources [Order Documents](http://www.nlm.nih.gov/loansomedoc/loansome_home.html) [NLM Catalog](http://www.ncbi.nlm.nih.gov/entrez/query.fcgi?db=nlmcatalog) [NLM Gateway](http://gateway.nlm.nih.gov/gw/Cmd) [TOXNET](http://toxnet.nlm.nih.gov/) [Consumer Health](http://www.nlm.nih.gov/medlineplus/) [Clinical Alerts](http://www.nlm.nih.gov/databases/alerts/clinical_alerts.html) [ClinicalTrials.gov](http://clinicaltrials.gov/ct/gui) [PubMed Central](http://www.pubmedcentral.nih.gov/) |  | | Field: **Title,** Limits: **All Child: 0-18 years, Publication Date to 2002** | | --- |  | Show: | | --- | |  |  | Items 1 - 18 of 18 | One page. | | --- | --- |  | **1:** | [Zuckerman GB, Lam SC, Santos SM.](http://www.ncbi.nlm.nih.gov/entrez/query.fcgi?cmd=Retrieve&db=pubmed&dopt=Abstract&list_uids=9490317) | [Related Articles,](http://www.ncbi.nlm.nih.gov/entrez/query.fcgi?db=pubmed&cmd=Display&dopt=pubmed_pubmed&from_uid=9490317) [Links](javascript:PopUpMenu2_Set(Menu9490317);) | | --- | --- | --- | |  | Rhabdomyolysis following oral ingestion of the hydrocarbon cyclohexanone in an adolescent. J Environ Pathol Toxicol Oncol. 1998;17(1):11-5. PMID: 9490317 [PubMed - indexed for MEDLINE] | |  | **2:** | [Anene O, Castello FV.](http://www.ncbi.nlm.nih.gov/entrez/query.fcgi?cmd=Retrieve&db=pubmed&dopt=Abstract&list_uids=8125006) | [Related Articles,](http://www.ncbi.nlm.nih.gov/entrez/query.fcgi?db=pubmed&cmd=Display&dopt=pubmed_pubmed&from_uid=8125006) [Links](javascript:PopUpMenu2_Set(Menu8125006);) | | --- | --- | --- | |  | Myocardial dysfunction after hydrocarbon ingestion. Crit Care Med. 1994 Mar;22(3):528-30. Review. No abstract available. PMID: 8125006 [PubMed - indexed for MEDLINE] | |  | **3:** | [Perrot LJ, Palmer H.](http://www.ncbi.nlm.nih.gov/entrez/query.fcgi?cmd=Retrieve&db=pubmed&dopt=Abstract&list_uids=1402767) | [Related Articles,](http://www.ncbi.nlm.nih.gov/entrez/query.fcgi?db=pubmed&cmd=Display&dopt=pubmed_pubmed&from_uid=1402767) [Links](javascript:PopUpMenu2_Set(Menu1402767);) | | --- | --- | --- | |  | Fatal hydrocarbon lipoid pneumonia and pneumonitis secondary to automatic transmission fluid ingestion. J Forensic Sci. 1992 Sep;37(5):1422-7. No abstract available. PMID: 1402767 [PubMed - indexed for MEDLINE] | |  | **4:** | [Vidal Company A, Barrio Merino A, Rodriguez Martin A, Garcia Lopez E.](http://www.ncbi.nlm.nih.gov/entrez/query.fcgi?cmd=Retrieve&db=pubmed&dopt=Abstract&list_uids=1741581) | [Related Articles,](http://www.ncbi.nlm.nih.gov/entrez/query.fcgi?db=pubmed&cmd=Display&dopt=pubmed_pubmed&from_uid=1741581) [Links](javascript:PopUpMenu2_Set(Menu1741581);) | | --- | --- | --- | |  | [Accidental ingestion of hydrocarbon and intravascular hemolysis: an infrequent complication] An Esp Pediatr. 1991 Sep;35(3):205-6. Spanish. No abstract available. PMID: 1741581 [PubMed - indexed for MEDLINE] | |  | **5:** | [Machado B, Cross K, Snodgrass WR.](http://www.ncbi.nlm.nih.gov/entrez/query.fcgi?cmd=Retrieve&db=pubmed&dopt=Abstract&list_uids=3394983) | [Related Articles,](http://www.ncbi.nlm.nih.gov/entrez/query.fcgi?db=pubmed&cmd=Display&dopt=pubmed_pubmed&from_uid=3394983) [Links](javascript:PopUpMenu2_Set(Menu3394983);) | | --- | --- | --- | |  | Accidental hydrocarbon ingestion cases telephoned to a regional poison center. Ann Emerg Med. 1988 Aug;17(8):804-7. PMID: 3394983 [PubMed - indexed for MEDLINE] | |  | **6:** | [Truemper E, Reyes de la Rocha S, Atkinson SD.](http://www.ncbi.nlm.nih.gov/entrez/query.fcgi?cmd=Retrieve&db=pubmed&dopt=Abstract&list_uids=3313305) | [Related Articles,](http://www.ncbi.nlm.nih.gov/entrez/query.fcgi?db=pubmed&cmd=Display&dopt=pubmed_pubmed&from_uid=3313305) [Links](javascript:PopUpMenu2_Set(Menu3313305);) | | --- | --- | --- | |  | Clinical characteristics, pathophysiology, and management of hydrocarbon ingestion: case report and review of the literature. Pediatr Emerg Care. 1987 Sep;3(3):187-93. Review. PMID: 3313305 [PubMed - indexed for MEDLINE] | |  | **7:** | [Tinker TD.](http://www.ncbi.nlm.nih.gov/entrez/query.fcgi?cmd=Retrieve&db=pubmed&dopt=Abstract&list_uids=3958820) | [Related Articles,](http://www.ncbi.nlm.nih.gov/entrez/query.fcgi?db=pubmed&cmd=Display&dopt=pubmed_pubmed&from_uid=3958820) [Links](javascript:PopUpMenu2_Set(Menu3958820);) | | --- | --- | --- | |  | Hydrocarbon ingestion in children: its sequelae and management. J Okla State Med Assoc. 1986 Feb;79(2):95-101. No abstract available. PMID: 3958820 [PubMed - indexed for MEDLINE] | |  | **8:** | [Marandian MH, Sabouri M, Youssefian H, Behvad A, Djafarian M.](http://www.ncbi.nlm.nih.gov/entrez/query.fcgi?cmd=Retrieve&db=pubmed&dopt=Abstract&list_uids=7316416) | [Related Articles,](http://www.ncbi.nlm.nih.gov/entrez/query.fcgi?db=pubmed&cmd=Display&dopt=pubmed_pubmed&from_uid=7316416) [Links](javascript:PopUpMenu2_Set(Menu7316416);) | | --- | --- | --- | |  | [Pneumatoceles and pneumothorax following accidental hydrocarbon ingestion in children. A study of 50 cases in Iran (author's transl)] Ann Pediatr (Paris). 1981 Nov;28(9):687-91. French. No abstract available. PMID: 7316416 [PubMed - indexed for MEDLINE] | |  | **9:** | [Marandian MH, Youssefian H, Saboury M, Haghigat H, Lessani M, Zaeri N.](http://www.ncbi.nlm.nih.gov/entrez/query.fcgi?cmd=Retrieve&db=pubmed&dopt=Abstract&list_uids=7305239) | [Related Articles,](http://www.ncbi.nlm.nih.gov/entrez/query.fcgi?db=pubmed&cmd=Display&dopt=pubmed_pubmed&from_uid=7305239) [Links](javascript:PopUpMenu2_Set(Menu7305239);) | | --- | --- | --- | |  | [Accidental hydrocarbon ingestion in children. Clinical, radiological, biological and pathological findings in 3,462 cases (author's transl)] Ann Pediatr (Paris). 1981 Oct;28(8):601-9. French. No abstract available. PMID: 7305239 [PubMed - indexed for MEDLINE] | |  | **10:** | [Mack RB.](http://www.ncbi.nlm.nih.gov/entrez/query.fcgi?cmd=Retrieve&db=pubmed&dopt=Abstract&list_uids=6945484) | [Related Articles,](http://www.ncbi.nlm.nih.gov/entrez/query.fcgi?db=pubmed&cmd=Display&dopt=pubmed_pubmed&from_uid=6945484) [Links](javascript:PopUpMenu2_Set(Menu6945484);) | | --- | --- | --- | |  | Hydrocarbon ingestion. N C Med J. 1981 May;42(5):338. No abstract available. PMID: 6945484 [PubMed - indexed for MEDLINE] | |  | **11:** | [Zieserl E.](http://www.ncbi.nlm.nih.gov/entrez/query.fcgi?cmd=Retrieve&db=pubmed&dopt=Abstract&list_uids=455933) | [Related Articles,](http://www.ncbi.nlm.nih.gov/entrez/query.fcgi?db=pubmed&cmd=Display&dopt=pubmed_pubmed&from_uid=455933) [Links](javascript:PopUpMenu2_Set(Menu455933);) | | --- | --- | --- | |  | Hydrocarbon ingestion and poisoning. Compr Ther. 1979 Jun;5(6):35-42. No abstract available. PMID: 455933 [PubMed - indexed for MEDLINE] | |  | **12:** | [**Beamon RF, Siegel CJ, Landers G, Green V.**](http://www.ncbi.nlm.nih.gov/entrez/query.fcgi?cmd=Retrieve&db=pubmed&dopt=Abstract&list_uids=1018351) | [Related Articles,](http://www.ncbi.nlm.nih.gov/entrez/query.fcgi?db=pubmed&cmd=Display&dopt=pubmed_pubmed&from_uid=1018351) [Links](javascript:PopUpMenu2_Set(Menu1018351);) | | --- | --- | --- | |  | **Hydrocarbon ingestion in children: a six-year retrospective study. JACEP. 1976 Oct;5(10):771-5.** PMID: 1018351 [PubMed - indexed for MEDLINE] | | |

- Paroxysmal supraventricular tachycardia:

| | [Entrez](http://www.ncbi.nlm.nih.gov/gquery/gquery.fcgi?itool=toolbar) | [PubMed](http://www.ncbi.nlm.nih.gov/entrez/query.fcgi?db=PubMed&itool=toolbar) | [Nucleotide](http://www.ncbi.nlm.nih.gov/entrez/query.fcgi?db=Nucleotide&itool=toolbar) | [Protein](http://www.ncbi.nlm.nih.gov/entrez/query.fcgi?db=Protein&itool=toolbar) | [Genome](http://www.ncbi.nlm.nih.gov/entrez/query.fcgi?db=Genome&itool=toolbar) | [Structure](http://www.ncbi.nlm.nih.gov/entrez/query.fcgi?db=Structure&itool=toolbar) | [OMIM](http://www.ncbi.nlm.nih.gov/entrez/query.fcgi?db=OMIM&itool=toolbar) | [PMC](http://www.ncbi.nlm.nih.gov/entrez/query.fcgi?db=PMC&itool=toolbar) | [Journals](http://www.ncbi.nlm.nih.gov/entrez/query.fcgi?db=Journals&itool=toolbar) | [Books](http://www.ncbi.nlm.nih.gov/entrez/query.fcgi?db=Books&itool=toolbar) | | --- | --- | --- | --- | --- | --- | --- | --- | --- | --- | |
| --- | --- | --- | --- | --- | --- | --- | --- | --- | --- | --- |
| | Search for | | --- | |
| |  | |  | [Limits](javascript:Go('Limits')) | [Preview/Index](javascript:Go('Index')) | [History](javascript:Go('History')) | [Clipboard](javascript:Go('Clipboard')) | [Details](javascript:Go('Details')) |  |  | | --- | --- | --- | --- | --- | --- | --- | --- | | | --- | --- | --- | --- | --- | --- | --- | --- | --- | --- | |

|  | | |
| --- | --- | --- |
| [About Entrez](http://www.ncbi.nlm.nih.gov/Database/index.html)  [Text Version](http://www.ncbi.nlm.nih.gov/entrez/queryd.fcgi?linkbar=plain)  Entrez PubMed  [Overview](http://www.ncbi.nlm.nih.gov/entrez/query/static/overview.html) [Help |](http://www.ncbi.nlm.nih.gov/entrez/query/static/help/pmhelp.html) [FAQ](http://www.ncbi.nlm.nih.gov/entrez/query/static/faq.html) [Tutorial](http://www.nlm.nih.gov/bsd/pubmed_tutorial/m1001.html) [New/Noteworthy](http://www.ncbi.nlm.nih.gov/entrez/query/static/new.html) [E-Utilities](http://eutils.ncbi.nlm.nih.gov/entrez/query/static/eutils_help.html)  PubMed Services [Journals Database](http://www.ncbi.nlm.nih.gov/entrez/query.fcgi?db=journals) [MeSH Database](http://www.ncbi.nlm.nih.gov/entrez/query.fcgi?db=mesh) [Single Citation Matcher](http://www.ncbi.nlm.nih.gov/entrez/query/static/citmatch.html) [Batch Citation Matcher](http://www.ncbi.nlm.nih.gov/entrez/getids.cgi) [Clinical Queries](http://www.ncbi.nlm.nih.gov/entrez/query/static/clinical.html) [LinkOut](http://www.ncbi.nlm.nih.gov/entrez/linkout) [Cubby](http://www.ncbi.nlm.nih.gov/entrez/cubby.fcgi?call=QueryExt.Query.last.Show&call=QueryExt.CubbyQuery..ShowAll)  Related Resources [Order Documents](http://www.nlm.nih.gov/loansomedoc/loansome_home.html) [NLM Catalog](http://www.ncbi.nlm.nih.gov/entrez/query.fcgi?db=nlmcatalog) [NLM Gateway](http://gateway.nlm.nih.gov/gw/Cmd) [TOXNET](http://toxnet.nlm.nih.gov/) [Consumer Health](http://www.nlm.nih.gov/medlineplus/) [Clinical Alerts](http://www.nlm.nih.gov/databases/alerts/clinical_alerts.html) [ClinicalTrials.gov](http://clinicaltrials.gov/ct/gui) [PubMed Central](http://www.pubmedcentral.nih.gov/) |  | | Field: **Title,** Limits: **Publication Date to 2002, Randomized Controlled Trial** | | --- |  | Show: | | --- | |  |  | Items 1 - 3 of 3 | One page. | | --- | --- |  | **1:** | [Ferreira JF, Pamplona D, Cesar LA, Leite PF, Sosa EA, da Luz PL, Bellotti G.](http://www.ncbi.nlm.nih.gov/entrez/query.fcgi?cmd=Retrieve&db=pubmed&dopt=Abstract&list_uids=8734859) | [Related Articles,](http://www.ncbi.nlm.nih.gov/entrez/query.fcgi?db=pubmed&cmd=Display&dopt=pubmed_pubmed&from_uid=8734859) [Links](javascript:PopUpMenu2_Set(Menu8734859);) | | --- | --- | --- | |  | [Comparative study between verapamil and adenosine triphosphate in the treatment of paroxysmal supraventricular tachycardia] Arq Bras Cardiol. 1996 Feb;66(2):55-7. Portuguese. PMID: 8734859 [PubMed - indexed for MEDLINE] | |  | **2:** | [Gil Madre J, Lazaro Rodriguez S, Sentenac Merchan G, Sepulveda Berrocal MA, Alises Moraleda JM, Cortes Bermejo S, Garcia de Pedro J, Lain Teres N.](http://www.ncbi.nlm.nih.gov/entrez/query.fcgi?cmd=Retrieve&db=pubmed&dopt=Abstract&list_uids=7878283) | [Related Articles,](http://www.ncbi.nlm.nih.gov/entrez/query.fcgi?db=pubmed&cmd=Display&dopt=pubmed_pubmed&from_uid=7878283) [Links](javascript:PopUpMenu2_Set(Menu7878283);) | | --- | --- | --- | |  | [Adenosine triphosphate in the treatment of supraventricular paroxysmal tachycardia: a comparison with verapamil] Rev Esp Cardiol. 1995 Jan;48(1):55-8. Spanish. PMID: 7878283 [PubMed - indexed for MEDLINE] | |  | **3:** | [DiMarco JP, Miles W, Akhtar M, Milstein S, Sharma AD, Platia E, McGovern B, Scheinman MM, Govier WC.](http://www.ncbi.nlm.nih.gov/entrez/query.fcgi?cmd=Retrieve&db=pubmed&dopt=Abstract&list_uids=2193560) | [Related Articles,](http://www.ncbi.nlm.nih.gov/entrez/query.fcgi?db=pubmed&cmd=Display&dopt=pubmed_pubmed&from_uid=2193560) [Links](javascript:PopUpMenu2_Set(Menu2193560);) | | --- | --- | --- | |  | Adenosine for paroxysmal supraventricular tachycardia: dose ranging and comparison with verapamil. Assessment in placebo-controlled, multicenter trials. The Adenosine for PSVT Study Group. Ann Intern Med. 1990 Jul 15;113(2):104-10.Erratum in: Ann Intern Med 1990 Dec 15;113(12):996.  PMID: 2193560 [PubMed - indexed for MEDLINE] | | |

- Aspiration pneumonia:

| | [Entrez](http://www.ncbi.nlm.nih.gov/gquery/gquery.fcgi?itool=toolbar) | [PubMed](http://www.ncbi.nlm.nih.gov/entrez/query.fcgi?db=PubMed&itool=toolbar) | [Nucleotide](http://www.ncbi.nlm.nih.gov/entrez/query.fcgi?db=Nucleotide&itool=toolbar) | [Protein](http://www.ncbi.nlm.nih.gov/entrez/query.fcgi?db=Protein&itool=toolbar) | [Genome](http://www.ncbi.nlm.nih.gov/entrez/query.fcgi?db=Genome&itool=toolbar) | [Structure](http://www.ncbi.nlm.nih.gov/entrez/query.fcgi?db=Structure&itool=toolbar) | [OMIM](http://www.ncbi.nlm.nih.gov/entrez/query.fcgi?db=OMIM&itool=toolbar) | [PMC](http://www.ncbi.nlm.nih.gov/entrez/query.fcgi?db=PMC&itool=toolbar) | [Journals](http://www.ncbi.nlm.nih.gov/entrez/query.fcgi?db=Journals&itool=toolbar) | [Books](http://www.ncbi.nlm.nih.gov/entrez/query.fcgi?db=Books&itool=toolbar) | | --- | --- | --- | --- | --- | --- | --- | --- | --- | --- | |
| --- | --- | --- | --- | --- | --- | --- | --- | --- | --- | --- |
| | Search for | | --- | |
| |  | |  | [Limits](javascript:Go('Limits')) | [Preview/Index](javascript:Go('Index')) | [History](javascript:Go('History')) | [Clipboard](javascript:Go('Clipboard')) | [Details](javascript:Go('Details')) |  |  | | --- | --- | --- | --- | --- | --- | --- | --- | | | --- | --- | --- | --- | --- | --- | --- | --- | --- | --- | |

|  | | |
| --- | --- | --- |
| [About Entrez](http://www.ncbi.nlm.nih.gov/Database/index.html)  [Text Version](http://www.ncbi.nlm.nih.gov/entrez/queryd.fcgi?linkbar=plain)  Entrez PubMed  [Overview](http://www.ncbi.nlm.nih.gov/entrez/query/static/overview.html) [Help |](http://www.ncbi.nlm.nih.gov/entrez/query/static/help/pmhelp.html) [FAQ](http://www.ncbi.nlm.nih.gov/entrez/query/static/faq.html) [Tutorial](http://www.nlm.nih.gov/bsd/pubmed_tutorial/m1001.html) [New/Noteworthy](http://www.ncbi.nlm.nih.gov/entrez/query/static/new.html) [E-Utilities](http://eutils.ncbi.nlm.nih.gov/entrez/query/static/eutils_help.html)  PubMed Services [Journals Database](http://www.ncbi.nlm.nih.gov/entrez/query.fcgi?db=journals) [MeSH Database](http://www.ncbi.nlm.nih.gov/entrez/query.fcgi?db=mesh) [Single Citation Matcher](http://www.ncbi.nlm.nih.gov/entrez/query/static/citmatch.html) [Batch Citation Matcher](http://www.ncbi.nlm.nih.gov/entrez/getids.cgi) [Clinical Queries](http://www.ncbi.nlm.nih.gov/entrez/query/static/clinical.html) [LinkOut](http://www.ncbi.nlm.nih.gov/entrez/linkout) [Cubby](http://www.ncbi.nlm.nih.gov/entrez/cubby.fcgi?call=QueryExt.Query.last.Show&call=QueryExt.CubbyQuery..ShowAll)  Related Resources [Order Documents](http://www.nlm.nih.gov/loansomedoc/loansome_home.html) [NLM Catalog](http://www.ncbi.nlm.nih.gov/entrez/query.fcgi?db=nlmcatalog) [NLM Gateway](http://gateway.nlm.nih.gov/gw/Cmd) [TOXNET](http://toxnet.nlm.nih.gov/) [Consumer Health](http://www.nlm.nih.gov/medlineplus/) [Clinical Alerts](http://www.nlm.nih.gov/databases/alerts/clinical_alerts.html) [ClinicalTrials.gov](http://clinicaltrials.gov/ct/gui) [PubMed Central](http://www.pubmedcentral.nih.gov/) |  | | Field: **Title,** Limits: **All Child: 0-18 years, Publication Date to 2002, Randomized Controlled Trial** | | --- |  | Show: | | --- | |  |  | Items 1 - 3 of 3 | One page. | | --- | --- |  | **1:** | [**Jacobson SJ, Griffiths K, Diamond S, Winders P, Sgro M, Feldman W, Macarthur C.**](http://www.ncbi.nlm.nih.gov/entrez/query.fcgi?cmd=Retrieve&db=pubmed&dopt=Abstract&list_uids=9232045) | [Related Articles,](http://www.ncbi.nlm.nih.gov/entrez/query.fcgi?db=pubmed&cmd=Display&dopt=pubmed_pubmed&from_uid=9232045) [Links](javascript:PopUpMenu2_Set(Menu9232045);) | | --- | --- | --- | |  | **A randomized controlled trial of penicillin vs clindamycin for the treatment of aspiration pneumonia in children. Arch Pediatr Adolesc Med. 1997 Jul;151(7):701-4.** PMID: 9232045 [PubMed - indexed for MEDLINE] | |  | **2:** | [Tulli G, Ciocca V, Nannoni S, Gabini R, De Gregori P, Casilini A, Roggi V, Sguerri D.](http://www.ncbi.nlm.nih.gov/entrez/query.fcgi?cmd=Retrieve&db=pubmed&dopt=Abstract&list_uids=3033549) | [Related Articles,](http://www.ncbi.nlm.nih.gov/entrez/query.fcgi?db=pubmed&cmd=Display&dopt=pubmed_pubmed&from_uid=3033549) [Links](javascript:PopUpMenu2_Set(Menu3033549);) | | --- | --- | --- | |  | [Ranitidine, cimetidine and magnesium silicate in the prevention of aspiration pneumonia] Minerva Anestesiol. 1986 Nov;52(11):375-83. Italian. No abstract available. PMID: 3033549 [PubMed - indexed for MEDLINE] | |  | **3:** | [Tryba M, Yildiz F, Weissenborn U, Hausdorfer J.](http://www.ncbi.nlm.nih.gov/entrez/query.fcgi?cmd=Retrieve&db=pubmed&dopt=Abstract&list_uids=6614410) | [Related Articles,](http://www.ncbi.nlm.nih.gov/entrez/query.fcgi?db=pubmed&cmd=Display&dopt=pubmed_pubmed&from_uid=6614410) [Links](javascript:PopUpMenu2_Set(Menu6614410);) | | --- | --- | --- | |  | [Efficacy of cimetidine in the prevention of aspiration pneumonia in paediatric anaesthesia] Anasth Intensivther Notfallmed. 1983 Jun;18(3):116-20. German. PMID: 6614410 [PubMed - indexed for MEDLINE] | | |

- Post infectious cerebellitis:

| | [Entrez](http://www.ncbi.nlm.nih.gov/gquery/gquery.fcgi?itool=toolbar) | [PubMed](http://www.ncbi.nlm.nih.gov/entrez/query.fcgi?db=PubMed&itool=toolbar) | [Nucleotide](http://www.ncbi.nlm.nih.gov/entrez/query.fcgi?db=Nucleotide&itool=toolbar) | [Protein](http://www.ncbi.nlm.nih.gov/entrez/query.fcgi?db=Protein&itool=toolbar) | [Genome](http://www.ncbi.nlm.nih.gov/entrez/query.fcgi?db=Genome&itool=toolbar) | [Structure](http://www.ncbi.nlm.nih.gov/entrez/query.fcgi?db=Structure&itool=toolbar) | [OMIM](http://www.ncbi.nlm.nih.gov/entrez/query.fcgi?db=OMIM&itool=toolbar) | [PMC](http://www.ncbi.nlm.nih.gov/entrez/query.fcgi?db=PMC&itool=toolbar) | [Journals](http://www.ncbi.nlm.nih.gov/entrez/query.fcgi?db=Journals&itool=toolbar) | [Books](http://www.ncbi.nlm.nih.gov/entrez/query.fcgi?db=Books&itool=toolbar) | | --- | --- | --- | --- | --- | --- | --- | --- | --- | --- | |
| --- | --- | --- | --- | --- | --- | --- | --- | --- | --- | --- |
| | Search for | | --- | |
| |  | |  | [Limits](javascript:Go('Limits')) | [Preview/Index](javascript:Go('Index')) | [History](javascript:Go('History')) | [Clipboard](javascript:Go('Clipboard')) | [Details](javascript:Go('Details')) |  |  | | --- | --- | --- | --- | --- | --- | --- | --- | | | --- | --- | --- | --- | --- | --- | --- | --- | --- | --- | |

|  | | |
| --- | --- | --- |
| [About Entrez](http://www.ncbi.nlm.nih.gov/Database/index.html)  [Text Version](http://www.ncbi.nlm.nih.gov/entrez/queryd.fcgi?linkbar=plain)  Entrez PubMed  [Overview](http://www.ncbi.nlm.nih.gov/entrez/query/static/overview.html) [Help |](http://www.ncbi.nlm.nih.gov/entrez/query/static/help/pmhelp.html) [FAQ](http://www.ncbi.nlm.nih.gov/entrez/query/static/faq.html) [Tutorial](http://www.nlm.nih.gov/bsd/pubmed_tutorial/m1001.html) [New/Noteworthy](http://www.ncbi.nlm.nih.gov/entrez/query/static/new.html) [E-Utilities](http://eutils.ncbi.nlm.nih.gov/entrez/query/static/eutils_help.html)  PubMed Services [Journals Database](http://www.ncbi.nlm.nih.gov/entrez/query.fcgi?db=journals) [MeSH Database](http://www.ncbi.nlm.nih.gov/entrez/query.fcgi?db=mesh) [Single Citation Matcher](http://www.ncbi.nlm.nih.gov/entrez/query/static/citmatch.html) [Batch Citation Matcher](http://www.ncbi.nlm.nih.gov/entrez/getids.cgi) [Clinical Queries](http://www.ncbi.nlm.nih.gov/entrez/query/static/clinical.html) [LinkOut](http://www.ncbi.nlm.nih.gov/entrez/linkout) [Cubby](http://www.ncbi.nlm.nih.gov/entrez/cubby.fcgi?call=QueryExt.Query.last.Show&call=QueryExt.CubbyQuery..ShowAll)  Related Resources [Order Documents](http://www.nlm.nih.gov/loansomedoc/loansome_home.html) [NLM Catalog](http://www.ncbi.nlm.nih.gov/entrez/query.fcgi?db=nlmcatalog) [NLM Gateway](http://gateway.nlm.nih.gov/gw/Cmd) [TOXNET](http://toxnet.nlm.nih.gov/) [Consumer Health](http://www.nlm.nih.gov/medlineplus/) [Clinical Alerts](http://www.nlm.nih.gov/databases/alerts/clinical_alerts.html) [ClinicalTrials.gov](http://clinicaltrials.gov/ct/gui) [PubMed Central](http://www.pubmedcentral.nih.gov/) |  | | Field: **Title/Abstract,** Limits: **Publication Date to 2002** | | --- |  | Show: | | --- | |  |  | Items 1 - 4 of 4 | One page. | | --- | --- |  | **1:** | [San Pedro EC, Mountz JM, Liu HG, Deutsch G.](http://www.ncbi.nlm.nih.gov/entrez/query.fcgi?cmd=Retrieve&db=pubmed&dopt=Abstract&list_uids=9554191) | [Related Articles,](http://www.ncbi.nlm.nih.gov/entrez/query.fcgi?db=pubmed&cmd=Display&dopt=pubmed_pubmed&from_uid=9554191) [Links](javascript:PopUpMenu2_Set(Menu9554191);) | | --- | --- | --- | |  | Postinfectious cerebellitis: clinical significance of Tc-99m HMPAO brain SPECT compared with MRI. Clin Nucl Med. 1998 Apr;23(4):212-6. PMID: 9554191 [PubMed - indexed for MEDLINE] | |  | **2:** | [Bakshi R, Bates VE, Kinkel PR, Mechtler LL, Kinkel WR.](http://www.ncbi.nlm.nih.gov/entrez/query.fcgi?cmd=Retrieve&db=pubmed&dopt=Abstract&list_uids=9543582) | [Related Articles,](http://www.ncbi.nlm.nih.gov/entrez/query.fcgi?db=pubmed&cmd=Display&dopt=pubmed_pubmed&from_uid=9543582) [Links](javascript:PopUpMenu2_Set(Menu9543582);) | | --- | --- | --- | |  | Magnetic resonance imaging findings in acute cerebellitis. Clin Imaging. 1998 Mar-Apr;22(2):79-85. PMID: 9543582 [PubMed - indexed for MEDLINE] | |  | **3:** | [**Daaboul Y, Vern BA, Blend MJ.**](http://www.ncbi.nlm.nih.gov/entrez/query.fcgi?cmd=Retrieve&db=pubmed&dopt=Abstract&list_uids=9471108) | [Related Articles,](http://www.ncbi.nlm.nih.gov/entrez/query.fcgi?db=pubmed&cmd=Display&dopt=pubmed_pubmed&from_uid=9471108) [Links](javascript:PopUpMenu2_Set(Menu9471108);) | | --- | --- | --- | |  | **Brain SPECT imaging and treatment with IVIg in acute post-infectious cerebellar ataxia: case report. Neurol Res. 1998 Jan;20(1):85-8.** PMID: 9471108 [PubMed - indexed for MEDLINE] | |  | **4:** | [Perez Carretero M, Poch Vinals R.](http://www.ncbi.nlm.nih.gov/entrez/query.fcgi?cmd=Retrieve&db=pubmed&dopt=Abstract&list_uids=7052747) | [Related Articles,](http://www.ncbi.nlm.nih.gov/entrez/query.fcgi?db=pubmed&cmd=Display&dopt=pubmed_pubmed&from_uid=7052747) [Links](javascript:PopUpMenu2_Set(Menu7052747);) | | --- | --- | --- | |  | [Vestibular manifestations in a case of acute post-infectious cerebellitis] An Otorrinolaringol Ibero Am. 1980;7(6):475-91. Spanish. No abstract available. PMID: 7052747 [PubMed - indexed for MEDLINE] | | |

- Idiopathic thrombocytopenic purpura:

| | [Entrez](http://www.ncbi.nlm.nih.gov/gquery/gquery.fcgi?itool=toolbar) | [PubMed](http://www.ncbi.nlm.nih.gov/entrez/query.fcgi?db=PubMed&itool=toolbar) | [Nucleotide](http://www.ncbi.nlm.nih.gov/entrez/query.fcgi?db=Nucleotide&itool=toolbar) | [Protein](http://www.ncbi.nlm.nih.gov/entrez/query.fcgi?db=Protein&itool=toolbar) | [Genome](http://www.ncbi.nlm.nih.gov/entrez/query.fcgi?db=Genome&itool=toolbar) | [Structure](http://www.ncbi.nlm.nih.gov/entrez/query.fcgi?db=Structure&itool=toolbar) | [OMIM](http://www.ncbi.nlm.nih.gov/entrez/query.fcgi?db=OMIM&itool=toolbar) | [PMC](http://www.ncbi.nlm.nih.gov/entrez/query.fcgi?db=PMC&itool=toolbar) | [Journals](http://www.ncbi.nlm.nih.gov/entrez/query.fcgi?db=Journals&itool=toolbar) | [Books](http://www.ncbi.nlm.nih.gov/entrez/query.fcgi?db=Books&itool=toolbar) | | --- | --- | --- | --- | --- | --- | --- | --- | --- | --- | |
| --- | --- | --- | --- | --- | --- | --- | --- | --- | --- | --- |
| | Search for | | --- | |
| |  | |  | [Limits](javascript:Go('Limits')) | [Preview/Index](javascript:Go('Index')) | [History](javascript:Go('History')) | [Clipboard](javascript:Go('Clipboard')) | [Details](javascript:Go('Details')) |  |  | | --- | --- | --- | --- | --- | --- | --- | --- | | | --- | --- | --- | --- | --- | --- | --- | --- | --- | --- | |

|  | | |
| --- | --- | --- |
| [About Entrez](http://www.ncbi.nlm.nih.gov/Database/index.html)  [Text Version](http://www.ncbi.nlm.nih.gov/entrez/queryd.fcgi?linkbar=plain)  Entrez PubMed  [Overview](http://www.ncbi.nlm.nih.gov/entrez/query/static/overview.html) [Help |](http://www.ncbi.nlm.nih.gov/entrez/query/static/help/pmhelp.html) [FAQ](http://www.ncbi.nlm.nih.gov/entrez/query/static/faq.html) [Tutorial](http://www.nlm.nih.gov/bsd/pubmed_tutorial/m1001.html) [New/Noteworthy](http://www.ncbi.nlm.nih.gov/entrez/query/static/new.html) [E-Utilities](http://eutils.ncbi.nlm.nih.gov/entrez/query/static/eutils_help.html)  PubMed Services [Journals Database](http://www.ncbi.nlm.nih.gov/entrez/query.fcgi?db=journals) [MeSH Database](http://www.ncbi.nlm.nih.gov/entrez/query.fcgi?db=mesh) [Single Citation Matcher](http://www.ncbi.nlm.nih.gov/entrez/query/static/citmatch.html) [Batch Citation Matcher](http://www.ncbi.nlm.nih.gov/entrez/getids.cgi) [Clinical Queries](http://www.ncbi.nlm.nih.gov/entrez/query/static/clinical.html) [LinkOut](http://www.ncbi.nlm.nih.gov/entrez/linkout) [Cubby](http://www.ncbi.nlm.nih.gov/entrez/cubby.fcgi?call=QueryExt.Query.last.Show&call=QueryExt.CubbyQuery..ShowAll)  Related Resources [Order Documents](http://www.nlm.nih.gov/loansomedoc/loansome_home.html) [NLM Catalog](http://www.ncbi.nlm.nih.gov/entrez/query.fcgi?db=nlmcatalog) [NLM Gateway](http://gateway.nlm.nih.gov/gw/Cmd) [TOXNET](http://toxnet.nlm.nih.gov/) [Consumer Health](http://www.nlm.nih.gov/medlineplus/) [Clinical Alerts](http://www.nlm.nih.gov/databases/alerts/clinical_alerts.html) [ClinicalTrials.gov](http://clinicaltrials.gov/ct/gui) [PubMed Central](http://www.pubmedcentral.nih.gov/) |  | | Field: **Title,** Limits: **All Child: 0-18 years, Publication Date to 2002, Practice Guideline** | | --- |  | Show: | | --- | |  |  | Items 1 - 4 of 4 | One page. | | --- | --- |  | **1:** | [De Mattia D, Del Principe D, Del Vecchio GC, Jankovic M, Arrighini A, Giordano P, Menichelli A, Mori P, Zecca M, Pession A.](http://www.ncbi.nlm.nih.gov/entrez/query.fcgi?cmd=Retrieve&db=pubmed&dopt=Abstract&list_uids=10756369) | [Related Articles,](http://www.ncbi.nlm.nih.gov/entrez/query.fcgi?db=pubmed&cmd=Display&dopt=pubmed_pubmed&from_uid=10756369) [Links](javascript:PopUpMenu2_Set(Menu10756369);) | | --- | --- | --- | |  | Acute childhood idiopathic thrombocytopenic purpura: AIEOP consensus guidelines for diagnosis and treatment.Associazione Italiana di Ematologia e Oncologia Pediatrica. Haematologica. 2000 Apr;85(4):420-4. Review. PMID: 10756369 [PubMed - indexed for MEDLINE] | |  | **2:** | [George JN, Woolf SH, Raskob GE.](http://www.ncbi.nlm.nih.gov/entrez/query.fcgi?cmd=Retrieve&db=pubmed&dopt=Abstract&list_uids=9556088) | [Related Articles,](http://www.ncbi.nlm.nih.gov/entrez/query.fcgi?db=pubmed&cmd=Display&dopt=pubmed_pubmed&from_uid=9556088) [Links](javascript:PopUpMenu2_Set(Menu9556088);) | | --- | --- | --- | |  | Idiopathic thrombocytopenic purpura: a guideline for diagnosis and management of children and adults. American Society of Hematology. Ann Med. 1998 Feb;30(1):38-44. PMID: 9556088 [PubMed - indexed for MEDLINE] | |  | **3:** | [**George JN, Woolf SH, Raskob GE, Wasser JS, Aledort LM, Ballem PJ, Blanchette VS, Bussel JB, Cines DB, Kelton JG, Lichtin AE, McMillan R, Okerbloom JA, Regan DH, Warrier I.**](http://www.ncbi.nlm.nih.gov/entrez/query.fcgi?cmd=Retrieve&db=pubmed&dopt=Abstract&list_uids=8704187) | [Related Articles,](http://www.ncbi.nlm.nih.gov/entrez/query.fcgi?db=pubmed&cmd=Display&dopt=pubmed_pubmed&from_uid=8704187) [Links](javascript:PopUpMenu2_Set(Menu8704187);) | | --- | --- | --- | |  | **Idiopathic thrombocytopenic purpura: a practice guideline developed by explicit methods for the American Society of Hematology. Blood. 1996 Jul 1;88(1):3-40. Review. No abstract available.** PMID: 8704187 [PubMed - indexed for MEDLINE] | |  | **4:** | [Eden OB, Lilleyman JS.](http://www.ncbi.nlm.nih.gov/entrez/query.fcgi?cmd=Retrieve&db=pubmed&dopt=Abstract&list_uids=1520013) | [Related Articles,](http://www.ncbi.nlm.nih.gov/entrez/query.fcgi?db=pubmed&cmd=Display&dopt=pubmed_pubmed&from_uid=1520013) [Links](javascript:PopUpMenu2_Set(Menu1520013);) | | --- | --- | --- | |  | Guidelines for management of idiopathic thrombocytopenic purpura. The British Paediatric Haematology Group. Arch Dis Child. 1992 Aug;67(8):1056-8. No abstract available. PMID: 1520013 [PubMed - indexed for MEDLINE] | | |

- Anal fissure:

| | [Entrez](http://www.ncbi.nlm.nih.gov/gquery/gquery.fcgi?itool=toolbar) | [PubMed](http://www.ncbi.nlm.nih.gov/entrez/query.fcgi?db=PubMed&itool=toolbar) | [Nucleotide](http://www.ncbi.nlm.nih.gov/entrez/query.fcgi?db=Nucleotide&itool=toolbar) | [Protein](http://www.ncbi.nlm.nih.gov/entrez/query.fcgi?db=Protein&itool=toolbar) | [Genome](http://www.ncbi.nlm.nih.gov/entrez/query.fcgi?db=Genome&itool=toolbar) | [Structure](http://www.ncbi.nlm.nih.gov/entrez/query.fcgi?db=Structure&itool=toolbar) | [OMIM](http://www.ncbi.nlm.nih.gov/entrez/query.fcgi?db=OMIM&itool=toolbar) | [PMC](http://www.ncbi.nlm.nih.gov/entrez/query.fcgi?db=PMC&itool=toolbar) | [Journals](http://www.ncbi.nlm.nih.gov/entrez/query.fcgi?db=Journals&itool=toolbar) | [Books](http://www.ncbi.nlm.nih.gov/entrez/query.fcgi?db=Books&itool=toolbar) | | --- | --- | --- | --- | --- | --- | --- | --- | --- | --- | |
| --- | --- | --- | --- | --- | --- | --- | --- | --- | --- | --- |
| | Search for | | --- | |
| |  | |  | [Limits](javascript:Go('Limits')) | [Preview/Index](javascript:Go('Index')) | [History](javascript:Go('History')) | [Clipboard](javascript:Go('Clipboard')) | [Details](javascript:Go('Details')) |  |  | | --- | --- | --- | --- | --- | --- | --- | --- | | | --- | --- | --- | --- | --- | --- | --- | --- | --- | --- | |

|  | | |
| --- | --- | --- |
| [About Entrez](http://www.ncbi.nlm.nih.gov/Database/index.html)  [Text Version](http://www.ncbi.nlm.nih.gov/entrez/queryd.fcgi?linkbar=plain)  Entrez PubMed  [Overview](http://www.ncbi.nlm.nih.gov/entrez/query/static/overview.html) [Help |](http://www.ncbi.nlm.nih.gov/entrez/query/static/help/pmhelp.html) [FAQ](http://www.ncbi.nlm.nih.gov/entrez/query/static/faq.html) [Tutorial](http://www.nlm.nih.gov/bsd/pubmed_tutorial/m1001.html) [New/Noteworthy](http://www.ncbi.nlm.nih.gov/entrez/query/static/new.html) [E-Utilities](http://eutils.ncbi.nlm.nih.gov/entrez/query/static/eutils_help.html)  PubMed Services [Journals Database](http://www.ncbi.nlm.nih.gov/entrez/query.fcgi?db=journals) [MeSH Database](http://www.ncbi.nlm.nih.gov/entrez/query.fcgi?db=mesh) [Single Citation Matcher](http://www.ncbi.nlm.nih.gov/entrez/query/static/citmatch.html) [Batch Citation Matcher](http://www.ncbi.nlm.nih.gov/entrez/getids.cgi) [Clinical Queries](http://www.ncbi.nlm.nih.gov/entrez/query/static/clinical.html) [LinkOut](http://www.ncbi.nlm.nih.gov/entrez/linkout) [Cubby](http://www.ncbi.nlm.nih.gov/entrez/cubby.fcgi?call=QueryExt.Query.last.Show&call=QueryExt.CubbyQuery..ShowAll)  Related Resources [Order Documents](http://www.nlm.nih.gov/loansomedoc/loansome_home.html) [NLM Catalog](http://www.ncbi.nlm.nih.gov/entrez/query.fcgi?db=nlmcatalog) [NLM Gateway](http://gateway.nlm.nih.gov/gw/Cmd) [TOXNET](http://toxnet.nlm.nih.gov/) [Consumer Health](http://www.nlm.nih.gov/medlineplus/) [Clinical Alerts](http://www.nlm.nih.gov/databases/alerts/clinical_alerts.html) [ClinicalTrials.gov](http://clinicaltrials.gov/ct/gui) [PubMed Central](http://www.pubmedcentral.nih.gov/) |  | | Field: **Title,** Limits: **All Child: 0-18 years, Publication Date to 2002, Randomized Controlled Trial** | | --- |  | Show: | | --- | |  |  | Items 1 - 8 of 8 | One page. | | --- | --- |  | **1:** | [Libertiny G, Knight JS, Farouk R.](http://www.ncbi.nlm.nih.gov/entrez/query.fcgi?cmd=Retrieve&db=pubmed&dopt=Abstract&list_uids=12463433) | [Related Articles,](http://www.ncbi.nlm.nih.gov/entrez/query.fcgi?db=pubmed&cmd=Display&dopt=pubmed_pubmed&from_uid=12463433) [Links](javascript:PopUpMenu2_Set(Menu12463433);) | | --- | --- | --- | |  | Randomised trial of topical 0.2% glyceryl trinitrate and lateral internal sphincterotomy for the treatment of patients with chronic anal fissure: long-term follow-up. Eur J Surg. 2002;168(7):418-21. PMID: 12463433 [PubMed - indexed for MEDLINE] | |  | **2:** | [Perrotti P, Bove A, Antropoli C, Molino D, Antropoli M, Balzano A, De Stefano G, Attena F.](http://www.ncbi.nlm.nih.gov/entrez/query.fcgi?cmd=Retrieve&db=pubmed&dopt=Abstract&list_uids=12432293) | [Related Articles,](http://www.ncbi.nlm.nih.gov/entrez/query.fcgi?db=pubmed&cmd=Display&dopt=pubmed_pubmed&from_uid=12432293) [Links](javascript:PopUpMenu2_Set(Menu12432293);) | | --- | --- | --- | |  | Topical nifedipine with lidocaine ointment vs. active control for treatment of chronic anal fissure: results of a prospective, randomized, double-blind study. Dis Colon Rectum. 2002 Nov;45(11):1468-75. PMID: 12432293 [PubMed - indexed for MEDLINE] | |  | **3:** | [**Sonmez K, Demirogullari B, Ekingen G, Turkyilmaz Z, Karabulut R, Basaklar AC, Kale N.**](http://www.ncbi.nlm.nih.gov/entrez/query.fcgi?cmd=Retrieve&db=pubmed&dopt=Abstract&list_uids=12194122) | [Related Articles,](http://www.ncbi.nlm.nih.gov/entrez/query.fcgi?db=pubmed&cmd=Display&dopt=pubmed_pubmed&from_uid=12194122) [Links](javascript:PopUpMenu2_Set(Menu12194122);) | | --- | --- | --- | |  | **Randomized, placebo-controlled treatment of anal fissure by lidocaine, EMLA, and GTN in children. J Pediatr Surg. 2002 Sep;37(9):1313-6.** PMID: 12194122 [PubMed - indexed for MEDLINE] | |  | **4:** | [Evans J, Luck A, Hewett P.](http://www.ncbi.nlm.nih.gov/entrez/query.fcgi?cmd=Retrieve&db=pubmed&dopt=Abstract&list_uids=11805569) | [Related Articles,](http://www.ncbi.nlm.nih.gov/entrez/query.fcgi?db=pubmed&cmd=Display&dopt=pubmed_pubmed&from_uid=11805569) [Links](javascript:PopUpMenu2_Set(Menu11805569);) | | --- | --- | --- | |  | Glyceryl trinitrate vs. lateral sphincterotomy for chronic anal fissure: prospective, randomized trial. Dis Colon Rectum. 2001 Jan;44(1):93-7. PMID: 11805569 [PubMed - indexed for MEDLINE] | |  | **5:** | [**Kenny SE, Irvine T, Driver CP, Nunn AT, Losty PD, Jones MO, Turnock RR, Lamont GL, Lloyd DA.**](http://www.ncbi.nlm.nih.gov/entrez/query.fcgi?cmd=Retrieve&db=pubmed&dopt=Abstract&list_uids=11668104) | [Related Articles,](http://www.ncbi.nlm.nih.gov/entrez/query.fcgi?db=pubmed&cmd=Display&dopt=pubmed_pubmed&from_uid=11668104) [Links](javascript:PopUpMenu2_Set(Menu11668104);) | | --- | --- | --- | |  | **Double blind randomised controlled trial of topical glyceryl trinitrate in anal fissure. Arch Dis Child. 2001 Nov;85(5):404-7.** PMID: 11668104 [PubMed - indexed for MEDLINE] | |  | **6:** | [Tander B, Guven A, Demirbag S, Ozkan Y, Ozturk H, Cetinkursun S.](http://www.ncbi.nlm.nih.gov/entrez/query.fcgi?cmd=Retrieve&db=pubmed&dopt=Abstract&list_uids=10626860) | [Related Articles,](http://www.ncbi.nlm.nih.gov/entrez/query.fcgi?db=pubmed&cmd=Display&dopt=pubmed_pubmed&from_uid=10626860) [Links](javascript:PopUpMenu2_Set(Menu10626860);) | | --- | --- | --- | |  | A prospective, randomized, double-blind, placebo-controlled trial of glyceryl-trinitrate ointment in the treatment of children with anal fissure. J Pediatr Surg. 1999 Dec;34(12):1810-2. PMID: 10626860 [PubMed - indexed for MEDLINE] | | |

- Kawasaki disease:

| | [Entrez](http://www.ncbi.nlm.nih.gov/gquery/gquery.fcgi?itool=toolbar) | [PubMed](http://www.ncbi.nlm.nih.gov/entrez/query.fcgi?db=PubMed&itool=toolbar) | [Nucleotide](http://www.ncbi.nlm.nih.gov/entrez/query.fcgi?db=Nucleotide&itool=toolbar) | [Protein](http://www.ncbi.nlm.nih.gov/entrez/query.fcgi?db=Protein&itool=toolbar) | [Genome](http://www.ncbi.nlm.nih.gov/entrez/query.fcgi?db=Genome&itool=toolbar) | [Structure](http://www.ncbi.nlm.nih.gov/entrez/query.fcgi?db=Structure&itool=toolbar) | [OMIM](http://www.ncbi.nlm.nih.gov/entrez/query.fcgi?db=OMIM&itool=toolbar) | [PMC](http://www.ncbi.nlm.nih.gov/entrez/query.fcgi?db=PMC&itool=toolbar) | [Journals](http://www.ncbi.nlm.nih.gov/entrez/query.fcgi?db=Journals&itool=toolbar) | [Books](http://www.ncbi.nlm.nih.gov/entrez/query.fcgi?db=Books&itool=toolbar) | | --- | --- | --- | --- | --- | --- | --- | --- | --- | --- | |
| --- | --- | --- | --- | --- | --- | --- | --- | --- | --- | --- |
| | Search for | | --- | |
| |  | |  | [Limits](javascript:Go('Limits')) | [Preview/Index](javascript:Go('Index')) | [History](javascript:Go('History')) | [Clipboard](javascript:Go('Clipboard')) | [Details](javascript:Go('Details')) |  |  | | --- | --- | --- | --- | --- | --- | --- | --- | | | --- | --- | --- | --- | --- | --- | --- | --- | --- | --- | |

|  | | |
| --- | --- | --- |
| [About Entrez](http://www.ncbi.nlm.nih.gov/Database/index.html)  [Text Version](http://www.ncbi.nlm.nih.gov/entrez/queryd.fcgi?linkbar=plain)  Entrez PubMed  [Overview](http://www.ncbi.nlm.nih.gov/entrez/query/static/overview.html) [Help |](http://www.ncbi.nlm.nih.gov/entrez/query/static/help/pmhelp.html) [FAQ](http://www.ncbi.nlm.nih.gov/entrez/query/static/faq.html) [Tutorial](http://www.nlm.nih.gov/bsd/pubmed_tutorial/m1001.html) [New/Noteworthy](http://www.ncbi.nlm.nih.gov/entrez/query/static/new.html) [E-Utilities](http://eutils.ncbi.nlm.nih.gov/entrez/query/static/eutils_help.html)  PubMed Services [Journals Database](http://www.ncbi.nlm.nih.gov/entrez/query.fcgi?db=journals) [MeSH Database](http://www.ncbi.nlm.nih.gov/entrez/query.fcgi?db=mesh) [Single Citation Matcher](http://www.ncbi.nlm.nih.gov/entrez/query/static/citmatch.html) [Batch Citation Matcher](http://www.ncbi.nlm.nih.gov/entrez/getids.cgi) [Clinical Queries](http://www.ncbi.nlm.nih.gov/entrez/query/static/clinical.html) [LinkOut](http://www.ncbi.nlm.nih.gov/entrez/linkout) [Cubby](http://www.ncbi.nlm.nih.gov/entrez/cubby.fcgi?call=QueryExt.Query.last.Show&call=QueryExt.CubbyQuery..ShowAll)  Related Resources [Order Documents](http://www.nlm.nih.gov/loansomedoc/loansome_home.html) [NLM Catalog](http://www.ncbi.nlm.nih.gov/entrez/query.fcgi?db=nlmcatalog) [NLM Gateway](http://gateway.nlm.nih.gov/gw/Cmd) [TOXNET](http://toxnet.nlm.nih.gov/) [Consumer Health](http://www.nlm.nih.gov/medlineplus/) [Clinical Alerts](http://www.nlm.nih.gov/databases/alerts/clinical_alerts.html) [ClinicalTrials.gov](http://clinicaltrials.gov/ct/gui) [PubMed Central](http://www.pubmedcentral.nih.gov/) |  | | Field: **Title,** Limits: **Publication Date to 2002, Meta-Analysis** | | --- |  | Show: | | --- | |  |  | Items 1 - 2 of 2 | One page. | | --- | --- |  | **1:** | [**Terai M, Shulman ST.**](http://www.ncbi.nlm.nih.gov/entrez/query.fcgi?cmd=Retrieve&db=pubmed&dopt=Abstract&list_uids=9427895) | [Related Articles,](http://www.ncbi.nlm.nih.gov/entrez/query.fcgi?db=pubmed&cmd=Display&dopt=pubmed_pubmed&from_uid=9427895) [Links](javascript:PopUpMenu2_Set(Menu9427895);) | | --- | --- | --- | |  | **Prevalence of coronary artery abnormalities in Kawasaki disease is highly dependent on gamma globulin dose but independent of salicylate dose.** **J Pediatr. 1997 Dec;131(6):888-93.** PMID: 9427895 [PubMed - indexed for MEDLINE] | |  | **2:** | [**Durongpisitkul K, Gururaj VJ, Park JM, Martin CF.**](http://www.ncbi.nlm.nih.gov/entrez/query.fcgi?cmd=Retrieve&db=pubmed&dopt=Abstract&list_uids=7491221) | [Related Articles,](http://www.ncbi.nlm.nih.gov/entrez/query.fcgi?db=pubmed&cmd=Display&dopt=pubmed_pubmed&from_uid=7491221) [Links](javascript:PopUpMenu2_Set(Menu7491221);) | | --- | --- | --- | |  | **The prevention of coronary artery aneurysm in Kawasaki disease: a meta-analysis on the efficacy of aspirin and immunoglobulin treatment. Pediatrics. 1995 Dec;96(6):1057-61.** PMID: 7491221 [PubMed - indexed for MEDLINE] | | |

- Painful crisis in sickle cell anaemia:

## Source: National Guideline Clearinghouse (http://www.guideline.gov/)

## Search Results

Your search criteria:
 
**Keyword:** *sickle cell*

Your search found 21 related guidelines, which are listed below.

To view a guideline summary, click on a title. The default view is the Brief Summary, from which you can also view the Complete Summary, XML View, Full Text, Palm Download, MS Word, Adobe PDF, or Guideline Synthesis by choosing the appropriate option in the Summary Box on the side menu.

To prepare a Guideline Comparison, add any of the guidelines listed to "My Collection" by selecting that guideline (check the box) and clicking the "Add to My Collection" button. For additional help, see [Guideline Comparison Help](http://www.guideline.gov/help/ConstructComparison.aspx).

Remember - Check the box next to a guideline to add it to "My Collection". Then click on the "Add to My Collection" button located on the page.

### Search Results:

The following guidelines were retrieved because they are linked to [concepts related to your query](http://www.guideline.gov/help/howtosearch.aspx#mapping) or because they [contain the words in your query](http://www.guideline.gov/help/howtosearch.aspx#mapping). Search results are listed in order of [relevance](http://www.guideline.gov/help/howtosearch.aspx#searchresults), unless otherwise specified in a Detailed Search.

| |  | **Title** | | --- | --- | |  |  | |  | [**Guideline for the management of acute and chronic pain in sickle cell disease.**](http://www.guideline.gov/summary/summary.aspx?doc_id=2621&nbr=1847&string=sickle+AND+cell)**American Pain Society - Professional Association.  1999 Aug.  96 pages.  NGC:001847** | |  |  | |  | [The management of priapism](http://www.guideline.gov/summary/summary.aspx?doc_id=3741&nbr=2967&string=sickle+AND+cell)  American Urological Association, Inc. - Medical Specialty Society.  2003.  Various pagings.  NGC:002967 | |  |  | |  | [Reduction of the influenza burden in children.](http://www.guideline.gov/summary/summary.aspx?doc_id=3527&nbr=2753&string=sickle+AND+cell)  American Academy of Pediatrics - Medical Specialty Society.  2002 Dec.  7 pages.  NGC:002753 | |  |  | |  | [Assessment: transcranial Doppler ultrasonography: report of the Therapeutics and Technology Assessment Subcommittee of the American Academy of Neurology.](http://www.guideline.gov/summary/summary.aspx?doc_id=5331&nbr=3644&string=sickle+AND+cell)  American Academy of Neurology - Medical Specialty Society.  2004 May 11 .  14 pages .  NGC:003644 | |  |  | |  | [Admission and discharge guidelines for the pediatric patient requiring intermediate care.](http://www.guideline.gov/summary/summary.aspx?doc_id=5090&nbr=3557&string=sickle+AND+cell)  American Academy of Pediatrics - Medical Specialty Society.  2004 May.  4 pages.  NGC:003557 | |
| --- | --- | --- | --- | --- | --- | --- | --- | --- | --- | --- | --- | --- | --- | --- | --- | --- | --- | --- | --- | --- | --- | --- |
